# Integrated single-cell transcriptome analysis reveals heterogeneity of esophageal squamous cell carcinoma microenvironment

**DOI:** 10.1038/s41467-021-27599-5

**Published:** 2021-12-17

**Authors:** Huy Q. Dinh, Feng Pan, Geng Wang, Qing-Feng Huang, Claire E. Olingy, Zhi-Yong Wu, Shao-Hong Wang, Xin Xu, Xiu-E Xu, Jian-Zhong He, Qian Yang, Sandra Orsulic, Marcela Haro, Li-Yan Li, Guo-Wei Huang, Joshua J. Breunig, H. Phillip Koeffler, Catherine C. Hedrick, Li-Yan Xu, De-Chen Lin, En-Min Li

**Affiliations:** 1 McArdle Laboratory for Cancer Research, University of Wisconsin-Madison School of Medicine and Public Health, Madison, WI USA; 2 Division of Inflammation Biology, La Jolla Institute for Immunology, La Jolla, CA USA; 3 Guangdong Provincial Key Laboratory of Infectious Diseases and Molecular Immunopathology, The Key Laboratory of Molecular Biology for High Cancer Incidence Coastal Chaoshan Area, Shantou University Medical College, Shantou, China; 4 Guangdong Esophageal Cancer Research Institute, Shantou Sub-center, Shantou, China; 5 Department of Thoracic Surgery, Cancer Hospital of Shantou University Medical College, Shantou, China; 6 Shantou Central Hospital, Shantou, China; 7 Department of Medicine, Samuel Oschin Comprehensive Cancer Institute, Cedars-Sinai Medical Center, Los Angeles, CA USA; 8 Department of Obstetrics and Gynecology and Samuel Oschin Comprehensive Cancer Institute, Cedars-Sinai Medical Center, David Geffen School of Medicine at UCLA, Los Angeles, CA USA; 9 Board of Governors Regenerative Medicine Institute and Department of Biomedical Sciences, Cedars-Sinai Medical Center, Los Angeles, CA USA

**Keywords:** Cancer microenvironment, Oesophageal cancer, Cancer genomics, Tumour heterogeneity, Transcriptomics

## Abstract

The tumor microenvironment is a highly complex ecosystem of diverse cell types, which shape cancer biology and impact the responsiveness to therapy. Here, we analyze the microenvironment of esophageal squamous cell carcinoma (ESCC) using single-cell transcriptome sequencing in 62,161 cells from blood, adjacent nonmalignant and matched tumor samples from 11 ESCC patients. We uncover heterogeneity in most cell types of the ESCC stroma, particularly in the fibroblast and immune cell compartments. We identify a tumor-specific subset of CST1^+^ myofibroblasts with prognostic values and potential biological significance. CST1^+^ myofibroblasts are also highly tumor-specific in other cancer types. Additionally, a subset of antigen-presenting fibroblasts is revealed and validated. Analyses of myeloid and T lymphoid lineages highlight the immunosuppressive nature of the ESCC microenvironment, and identify cancer-specific expression of immune checkpoint inhibitors. This work establishes a rich resource of stromal cell types of the ESCC microenvironment for further understanding of ESCC biology.

## Abstract (web summary)

The microenvironment of oesophageal squamous cell carcinomas (ESCC) is heterogeneous and can strongly impact response to treatment. Here, the authors characterize the ESCC tumour microenvironment with single-cell RNA-seq, finding CST1 + myofibroblasts with potential biological and prognostic significance as well as immunosuppression signatures.

## Introduction

Esophageal squamous cell carcinoma (ESCC) is common (>400,000 cases per year) and highly aggressive (causing ~300,000 deaths every year) malignancy^1^; ESCC patients have a 5-year survival rate lower than 20%^1^. Recently, immune checkpoint blockade therapy has shown encouraging efficacy in ESCC patients^2–4^. However, as with most solid tumors, only a minority of ESCC patients (20-30%) benefit from anti-PD-1 therapy^2–4^, highlighting that the immune system can be exploited for clinical benefits in ESCC. The variation in treatment efficacy of immune checkpoint blockade therapy has been associated with heterogeneity in the immune cell composition of individual tumors. Clearly, a better understanding of immune cell biology within the ESCC microenvironment will help identify mechanisms underlying cellular responsiveness or resistance to immunotherapies.

In addition to immune cells, multiple other cell types contribute to either resistance to immunotherapies or immune evasion, such as fibroblasts^5^, endothelial cells^6^ as well as cancer cells themselves. Indeed, we^7,8^ and others^9–11^ have reported that ESCC exhibits a high degree of intratumoral heterogeneity, which is notably associated with patient survival^11^. These prior results indicate that within the ESCC microenvironment, both stromal (including T cells, macrophages, fibroblasts, etc.) and malignant cells are highly heterogeneous. Moreover, the plethora of cell types and their distinct cellular states not only influence the immune system but also contribute to shape the biology of cancer cells, including their capabilities to survive, proliferate, migrate, metastasize, etc. However, the extent of cellular heterogeneity, the dynamics of distinct biological states, as well as their functional impact on the tumor ecosystem, remain largely uncharacterized in ESCC.

Single-cell transcriptome sequencing (scRNA-Seq) profiles gene expression network at the single-cell level, enabling high-resolution characterization of cellular heterogeneity, development, and differentiation states in diverse systems. This approach has been applied to analyze the tumor microenvironment of multiple cancer types, including breast cancer^12^, lung cancer^13^, hepatocellular cancer^14^, head and neck cancer^15^, pancreatic ductal adenocarcinoma (PDAC)^16^, etc. We have also recently revealed cellular heterogeneity using scRNA-Seq in both mouse and human glioma samples^17^.

Here, we perform scRNA-Seq to analyze the microenvironment from tumor and adjacent nonmalignant esophageal tissues from 11 ESCC patients. We reveal profound cellular heterogeneity of both lymphoid and myeloid cell lineages, highlighting an immunosuppressive ecosystem of ESCC tumors. In addition, we uncover prominent diversity of fibroblast compartment and identify a subset of CST1^+^ myofibroblasts with potential biological significance and prominent prognostic value. These results shed insights into esophageal cancer biology and provide important theoretical basis for advancing the therapeutic intervention for this malignancy.

## Results

### The cellular microenvironment of ESCC and adjacent nonmalignant esophageal tissues

**Fig. 1:**
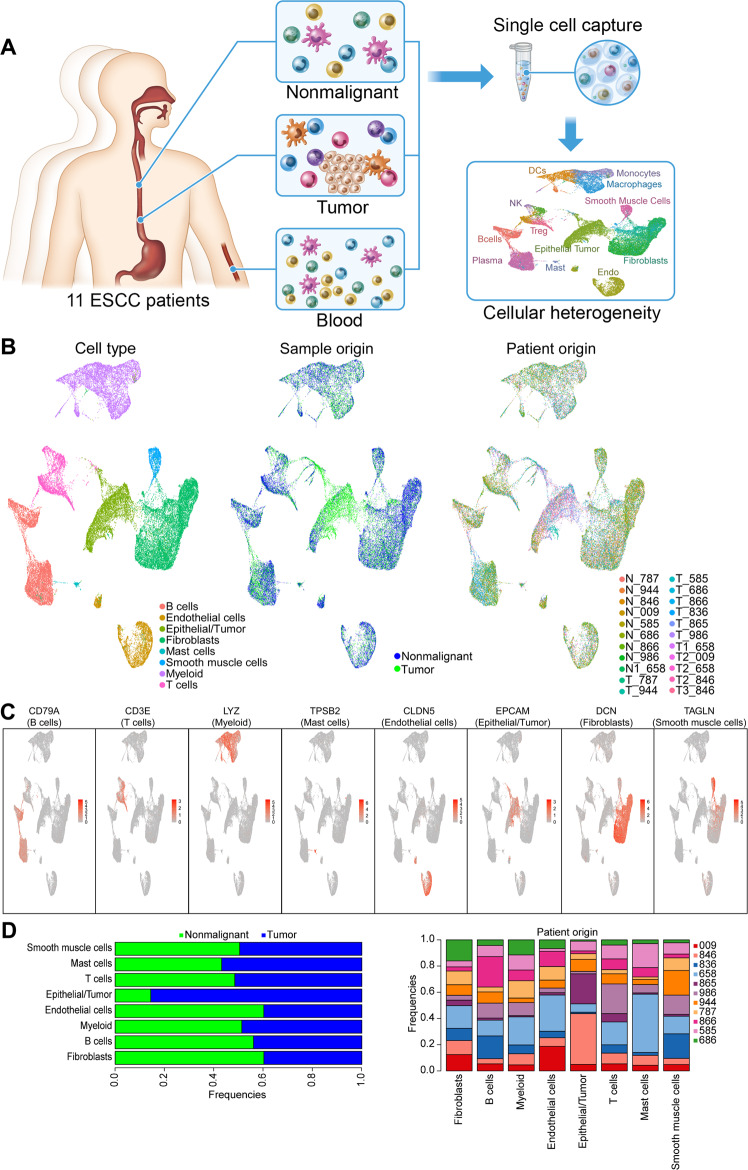
Single-cell transcriptomic landscape of esophageal squamous cell carcinoma (ESCC). **A** A schematic graph showing the study design. **B** UMAP (Uniform Manifold Approximation and Projection) visualization of the clustering of 41,237 cells from all 22 nonmalignant and tumor samples, color coded by either major cell type (left), sample type (middle) or patient origin (right). **C** Overlay of expression of representative marker genes for each cell type defined in (**B**). **D** The frequency of each cell type in nonmalignant and tumor samples (left), and in each of the 11 patients (right, an analysis restricted within tumor samples). Source data are provided as a Source Data file.

To explore the cellular heterogeneity in ESCC, we performed scRNA-Seq on primary tumors and matched adjacent nonmalignant esophageal tissues from 11 treatment-naive ESCC patients (Supplementary Table 1) using a droplet-based system that enables 3′ mRNA counting (10X Genomics Platform). Peripheral blood mononuclear cells (PBMCs) from three of the patients were also analyzed by scRNA-Seq (Fig. 1A). After single-cell capture and sequencing QC (see Methods), a total of 21,355 cells were obtained from ESCC tumors, 19,882 from nonmalignant esophagus and 20,924 cells from PBMC samples (Fig. 1A–B).

Because gene expression of tumor and nonmalignant cells is fundamentally different at the single-cell resolution^12,13^, we first analyzed 22 tissues samples (9 nonmalignant esophageal samples and 13 tumors) using a computational integration method. Specifically, to harmonize a single reference atlas of single-cell tumor and nonmalignant tissues, we employed an anchor-based pairwise integration method^18^ with each anchor representing a similar pair of cells from different samples (see Methods). The integration framework allowed us to account for the potential batch effect, sample variation, and in particular, to find shared biological states between nonmalignant and tumor samples while considering distinct subsets of each cell type. The integrated expression was used for further downstream analysis including dimensional reduction and cell type clustering. Cell type annotation and differential expression analysis were performed using raw RNA expression values before integration.

Subsequently, we performed enrichment scoring based on established markers for known cell types (see Methods). Through this approach, we identified epithelial cells (including both nonmalignant esophageal epithelial cells and tumor cells, termed Epithelial/Tumor), immune cells (myeloid, mast, T, and B cells), fibroblasts, smooth muscle cells, and endothelial cells (Fig. 1C; Supplementary Fig. 1–2). Most cell types were contributed comparably by tumor and nonmalignant samples, except for the “Epithelial/Tumor” compartment which was expectedly constituted by predominantly tumor samples (Fig. 1D). We also performed cell clustering using an independent approach (the CellRanger aggr method from 10X Genomics), and the data were presented in Supplementary Figs. 3–5.

### Fibroblast cell heterogeneity in ESCC

**Fig. 2:**
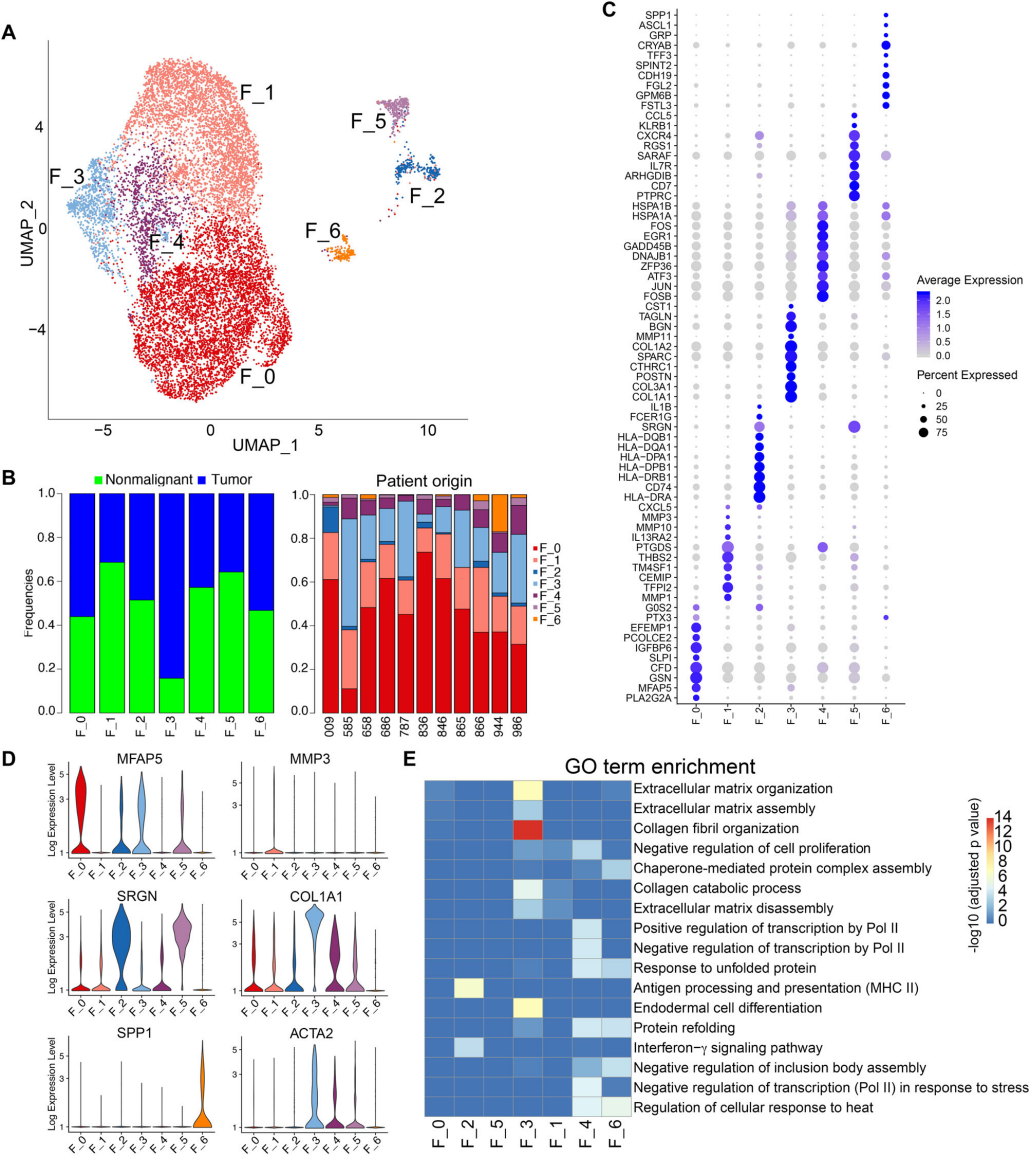
Fibroblast heterogeneity in ESCC. **A** UMAP visualization of the clustering of 12,126 fibroblast cells from all 22 nonmalignant and tumor samples. **B** The fraction of each fibroblast subset in nonmalignant and tumor samples (left), and in each of the 11 patients (right, an analysis restricted within tumor samples). **C** Dotplot showing the expression of top 10 most variable genes across each fibroblast subset. **D** Violin plots of the expression of representative ECM (extracellular matrix) genes and ACTA2 in each fibroblast subset. **E** Enrichment of GO (gene ontology) terms of each fibroblast subset (FDR-adjusted *P* < 0.001, Fisher exact test with multiple comparisons using topGO). Source data are provided as a Source Data file.

From nonmalignant and tumor samples, we detected a total of 12,126 fibroblast cells (Fig. 1B). To better understand fibroblast cell diversity within ESCC, we performed integrated analysis restricted on this compartment across nonmalignant and tumor samples and identified 7 subclusters (Fig. 2A). Common fibroblast markers such as DCN, FSP1 (also called S100A4), and mesenchymal cell marker VIM were expressed across all subpopulations (Supplementary Fig. 6A), confirming their fibroblastic cell identity. Additional markers were noted, such as SPARCL1 and PDPN (Supplementary Fig. 6A), both of which were found to be expressed by fibroblasts in other types of tissues^16,19,20^. Except for F_3 subset which was markedly enriched in tumors, all the other subsets had comparable abundance in both tumor and nonmalignant samples (Fig. 2B).

We next determined differentially expressed genes for each fibroblast subset using differential expression analysis of one subset in comparison to the rest (see Methods). One notable pattern emerged from the most variable genes was that different subsets expressed distinct repertoire of extracellular matrix (ECM) proteins (Fig. 2C–D, Supplementary Fig. 6A, Supplementary Data 1A), such as MFAP5 (F_0), MMP1, MMP3, MMP10 (F_1), SRGN (F_2 and F_5), COL1A1, COL3A1, POSTN, MMP11 (F_3), and SPP1 (F_6). Indeed, each subset of fibroblasts expressed at least one specific ECM gene, except for F_4 (Supplementary Data 1A). One of the most important functions of fibroblasts is to shape tissue structure by secreting molecules to remodel ECM, including collagen, MMPs, Laminin, Periostin, etc^5^. Thus, these results suggest functional specialization of different fibroblast populations in ESCC.

### Tumor-specific CST1^+^ myofibroblast is associated with poor prognosis in ESCC

**Fig. 3:**
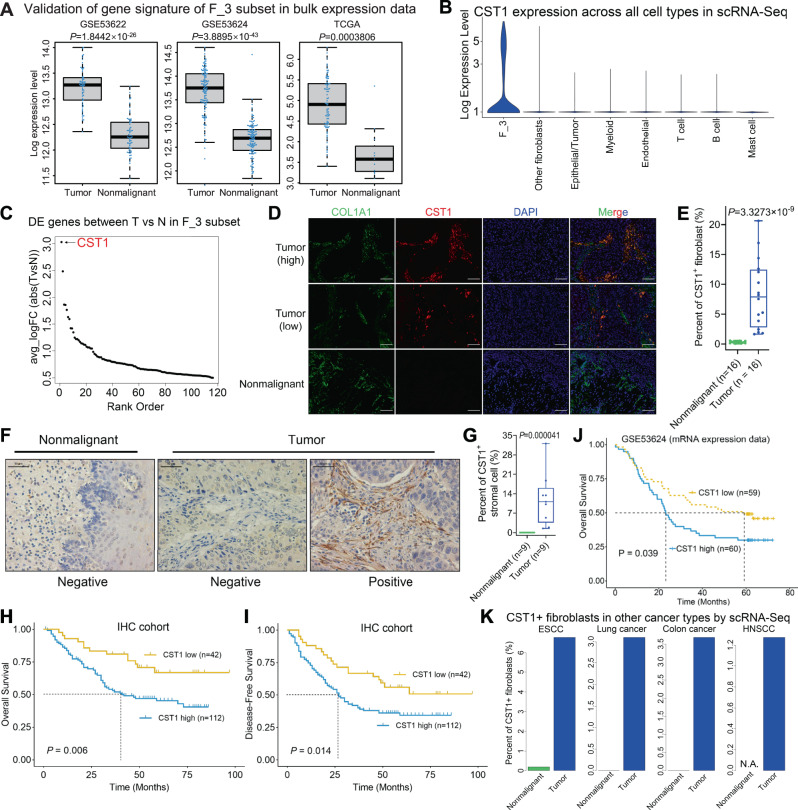
CST1^+^ myofibroblasts are cancer-specific and predict survival outcome in ESCC. **A** Box plots of the average levels of gene signature defined for F_3 in the TCGA bulk RNA-Seq and independent microarray datasets of ESCC samples. *n* = 60 paired tumor and nonmalignant samples in GSE53622, *n* = 119 paired tumor and nonmalignant samples in GSE53624, *n* = 80 tumor and 11 nonmalignant samples in TCGA. *P* values are calculated by two-tailed *t* test. **B** The expression of CST1 mRNA across myofibroblast (F_3), other fibroblasts and all the other cell types identified in ESCC. **C** Rank order of DE (differentially expressed) genes based on average logFC between tumor and nonmalignant samples within myofibroblasts; CST1 was identified as the most upregulated gene in tumor samples. N, nonmalignant; T, tumor. **D** Representative images of immunofluorescence double staining of both CST1 and COL1A1 in ESCC tumor and nonmalignant samples. Scale bar = 100 μm. **E** Quantification of the ratio of CST1^+^ cells out of COL1A1^+^ fibroblasts. **F** Representative images of IHC (immunohistochemistry) staining of CST1 in ESCC tumor and nonmalignant samples. Scale bar = 50 μm. **G** Quantification of the percent of CST1^+^ fibroblasts out of all stromal cells from IHC staining. **H** Kaplan-Meier curves of either overall survival or (**I**) disease-free survival of ESCC patients stratified by the abundance of CST1^+^ fibroblasts. **J** Kaplan-Meier curve of overall survival of ESCC patients stratified by the mRNA level of CST1 in an independent cohort. **K** Bar plots of the percentages of cells expressing CST1 mRNA in different scRNA-Seq datasets for different cancer types: ESCC (this study), lung, colon and head and neck. N.A., no data available from nonmalignant samples. In the box plots (**A**, **E**, **G**), the middle bar represents the median, and the box represents the interquartile range; whiskers indicate the maximum and minimum values. Dots are all the data points including outliers. *P* values are calculated by two-tailed Mann Whitney U test. Source data are provided as a Source Data file.

To gain further insights into the functionality of different subsets of fibroblasts, we performed Gene Ontology (GO) analysis based on the differentially expressed genes of each subset (see Methods). The most significantly enriched Biological Process GO terms were “Collagen fibril organization”, “ECM organization” and “Endodermal cell differentiation” in F_3 subset (Fig. 2E). Indeed, the majority of the ECM genes were expressed at the highest levels in F_3 compared with other subsets (Supplementary Fig. 6A). Furthermore, multiple lines of evidence together suggest that F_3 subset represents activated myofibroblasts: i) αSMA (encoded by ACTA2), a well-known myofibroblast marker, was most robustly expressed in F_3 (Fig. 2D); ii) Hallmark pathway analysis indicated that F_3 was highly proliferative, displaying the highest score of Mitotic spindle (Supplementary Fig. 6B); iii) F_3 showed the strongest protein secretion pathway (Supplementary Fig. 6B); iv) both EMT (epithelial-mesenchymal transition) and TGF-beta signatures had the highest scores in F_3 (Supplementary Fig. 6B). In several tumor types, myofibroblasts have been shown to promote cancer development and progression through various avenues^5,16,21^. Consistent with our scRNA-Seq analysis that F_3 was considerably more abundant in ESCC tumor than nonmalignant samples (Fig. 2B), F_3 gene signature was markedly stronger in tumors in comparison to nonmalignant samples across large ESCC cohorts of bulk expression datasets such as TCGA (Fig. 3A).

We next sought to identify specific markers for validation of this subset of activated myofibroblasts in ESCC. We performed differential expression analysis between tumor and nonmalignant samples within F_3 cluster and identified CST1 as the top cancer-specific gene (average logFC=3.0, Fig. 3C). The upregulation of CST1 in ESCC tumors was readily validated in bulk expression datasets (Supplementary Fig. 7A). In addition, CST1 was also among the top 10 most differentially expressed genes in F_3 compared with other fibroblast subsets (Fig. 2C). Strikingly, CST1 marked specifically F_3 myofibroblast not only across all fibroblast subsets but also across all the other cell types in our samples (Fig. 3B).

We next performed immunofluorescence (IF) double-staining using specific antibodies against CST1 and COL1A1 in 16 tumors and 16 matched nonmalignant esophagus samples from ESCC patients. We confirmed that CST1 protein was expressed in a subset (8.31%) of COL1A1^+^ fibroblasts in tumor samples, but it was barely detectable (0.27%) in nonmalignant samples (Fig. 3D–E). In another 9 nonmalignant/tumor paired ESCC cases, immunohistochemistry (IHC) results validated the prominent CST1 protein expression in the stroma of tumor-associated fibroblasts (11.95%), while it was absent in adjacent nonmalignant esophagus (Fig. 3F–G). Extending the IHC staining to a total of 154 ESCC patients (Supplementary Table 2), we confirmed the prevalence of CST1^+^ myofibroblasts in the tumor stromal of ESCC patients. Importantly, higher abundance of CST1^+^ myofibroblasts was strongly correlated with both shorter overall survival (*P* = 0.0051, Fig. 3H) and disease-free survival (*P* = 0.014, Fig. 3I). Moreover, multivariate Cox regression analysis identified CST1^+^ myofibroblasts as an independent prognostic marker (*P* = 0.041) for ESCC patients (Supplementary Table 3). In addition, a significant correlation was observed between CST1 expression and lymph node metastasis (*P* = 0.023), invasion depth (*P* < 0.001) and pTNM-stage (*P* < 0.001, Supplementary Table 4). The prognostic value of CST1 expression was verified in another cohort of ESCC patients using bulk expression data (*P* = 0.039, Fig. 3J). These data together highlight the prognostic value of CST1^+^ myofibroblasts in ESCC and indicate their potential biological significance.

To explore the presence of CST1^+^ myofibroblasts in other cancer types, we re-analyzed publicly-available single-cell transcriptomes which contained fibroblasts. Indeed, we observed CST1 expression in a subset of fibroblasts from lung, colon, and head and neck cancers with comparable frequencies (1.22–3.61%, Fig. 3K). Moreover, consistent with our data in ESCC, CST1^+^ fibroblasts were only detected in tumor samples from these cancer types, again confirming their cancer-specificity.

### Antigen presentation fibroblasts (ap-Fibro) in ESCC

**Fig. 4:**
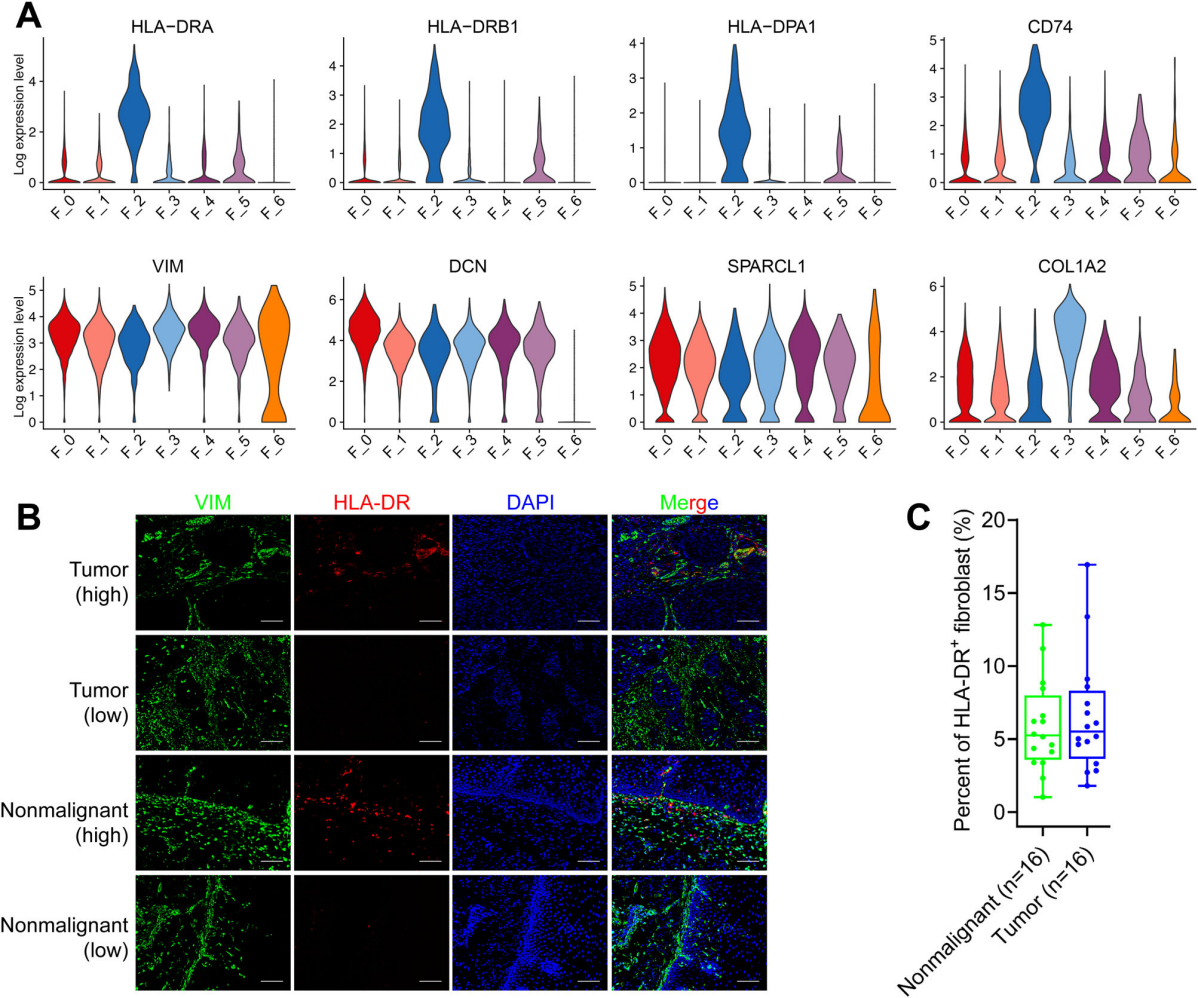
Antigen-presentation fibroblast in ESCC. **A** Violin plots of the expression of MHC II class genes and selected fibroblast marker genes in each fibroblast subset. **B** Representative images of immunofluorescence double staining of both HLA-DR and VIM in ESCC tumor and nonmalignant samples. Scale bar = 100 μm. **C** Quantification of the ratio of HLA-DR^+^ cells out of VIM^+^ fibroblasts. The middle bar represents the median, and the box represents the interquartile range; whiskers indicate the maximum and minimum values. Source data are provided as a Source Data file.

Another subset F_2 interestingly exhibited specific enrichment of GO terms “Antigen processing and presentation” and “Interferon-gamma signaling pathway” (Fig. 2E). Concordantly, all top differentially expressed genes in F_2 belonged to the MHC class II (e.g., HLA-DRA, CD74, HLA-DRB1, HLA-DPB1, Fig. 2C, Fig. 4A). These genes are typically restricted to antigen-presenting cells (APC) such as dendritic cells (DCs) and macrophages, and therefore, we termed these F_2 cells “antigen-presentation fibroblasts” (ap-Fibro). Antigen-presentation fibroblast was identified recently in both pancreatic cancer^16^ and normal pancreas^22^. Consistent with GO enrichment results, Hallmark pathway analysis showed that APC-related functions, such as allograft rejection and interferon-gamma response, were enriched highly in ap-Fibro cells (Supplementary Fig. 6B). Ap-Fibro also expressed well-defined fibroblast markers (DCN, VIM, FSP1) at similar levels with other subsets of fibroblasts (Fig. 4A, Supplementary Fig. 6A), confirming that they were cells with fibroblastic characteristics. In addition to MHC II genes, ap-Fibro also expressed IL1B (Fig. 2C), a cytokine often secreted by activated fibroblasts^5^.

To confirm the presence of ap-Fibro in ESCC tissues, we performed IF double-staining of both VIM and HLA-DR on cancerous and matched nonmalignant samples from 16 ESCC patients. Importantly, HLA-DR was confirmed to be expressed in a subset of VIM^+^ cells (Fig. 4B). Moreover, the fraction of HLA-DR^+^ fibroblasts determined by IF staining (5.87% in nonmalignant samples, 6.53% in tumor samples, Fig. 4C) was comparable to that determined by scRNA-Seq (2.2% in nonmalignant samples and 3.0% in tumors). This result was further confirmed by multiplexed IF staining using a panel of three antibodies (CD45, HLA-DR, VIM) on three matched ESCC tumor and nonmalignant samples (Supplementary Fig. 8).

### Immune cell microenvironment of ESCC

**Fig. 5:**
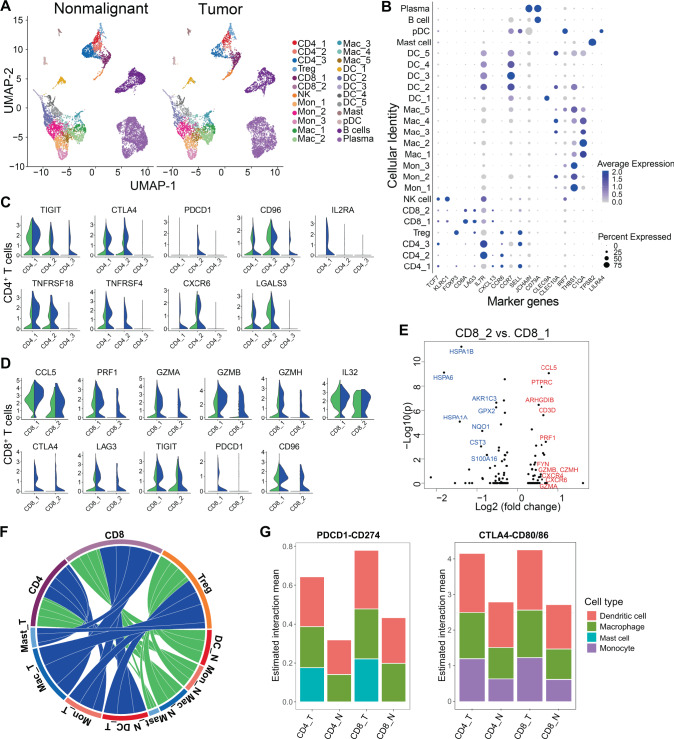
Myeloid and T cell landscapes of ESCC. **A** UMAP visualization of 17,923 immune cells extracted in silico from all 22 nonmalignant and tumor samples. **B** Dotplot showing the average expression of representative marker genes and percentages of expressed cells in each immune subset. **C** Violin plots showing the expression of selected genes for CD4^+^ T cell subsets or (**D**) CD8^+^ T cell subsets across nonmalignant (green) and tumor (blue) samples. **E** Volcano plot of the differentially expressed genes between the two CD8^+^ T cell subsets (CD8_1 and CD8_2 specific genes are highlighted in blue and red, respectively). **F** A circos plot showing the higher overall number of significant interacting pairs estimated by CellPhoneDB (*P* < 0.05) between myeloid and T cell subsets in tumor (blue) and nonmalignant (green) samples. **G** Estimated mean interaction scores for specific interactions (PDCD1-CD274, CTLA4-CD80/CD86) from indicated cell types in tumor and nonmalignant samples. N, nonmalignant; T, tumor. Source data are provided as a Source Data file.

We identified a total of 10,131 and 7,792 immune cells respectively from nonmalignant and tumor samples (Fig. 1B). Integrative analysis restricted on all immune cells revealed common references for the distinct immune population in both the tumor and nonmalignant samples, including T and NK cells, myeloid and mast cells, B and plasma cells (Fig. 5A). Further clustering analysis for T cell and myeloid compartments based on defined markers (see Methods) led to the identification of four CD4^+^ and two CD8^+^ T cell subsets, eight monocyte/macrophage subsets, and six dendritic cell (DC) subsets in most of the patients from both tumor and nonmalignant tissues (Fig. 5A–B, Supplementary Fig. 9).

We identified three clusters of CD4^+^ T cells, in addition to T regulatory cells (Treg, based on the expression of FOXP3). Consistent with other cancers such as PDAC^16^ and lung cancer^13^, Treg cells were more accumulated in ESCC tumors (Supplementary Fig. 9A, C). To explore the biological features of these CD4^+^ T cell subsets, we determined gene signatures for each subset. Of the other three subsets of CD4^+^ T cells, CD4_1 expressed genes which are abundant in Follicular helper T (TFH) effectors (CXCL13, IL2RA, TNFRSF18, TNFRSF, Fig. 5C, Supplementary Data 1B). CD4_2 subset showed effector memory gene signature, including IL7R, CXCR6, and Galectin-3 (encoded by LGALS3), and it was more abundant in nonmalignant samples. Notably, different CD4^+^ T cell subsets expressed distinct immune checkpoint factors in ESCC tumors compared to nonmalignant samples CD4_1 had upregulated TIGIT expression in tumors, CD4_2 expressed PD-1 (encoded by PDCD1) exclusively in tumor samples, CD4_3 showed tumor-specific TIGIT and CD96 expression^23^. In addition, both CD4_1 and CD4_2 expressed higher CTLA-4 in tumors (Fig. 5C).

Both subsets of CD8^+^ T cells from tumor samples expressed higher effector genes than nonmalignant samples, including chemokines (e.g., CCL5), cytotoxicity-associated genes (PRF1, GZMA, GZMB, GZMH), and proinflammatory cytokines (e.g., IL-32) (Fig. 5D, Supplementary Data 1C). Both clusters also exhibited a cancer-specific exhaustive signature characterized by higher expression of checkpoint molecules (CTLA-4, LAG3, TIGIT). In addition, CD8_1 and CD8_2 had respectively increased levels of PD-1 and CD96 in tumor samples (Fig. 5D). These results suggest that the effector function of CD8^+^ T cells is curtailed by co-inhibitory factors in the tumor microenvironment of ESCC. Moreover, comparing the two CD8^+^ T cell subsets revealed distinct gene signatures (Fig. 5E): CD8_1 expressed higher effector markers, including CCL5, CXCR4, CXCR6, PRF1, GZMB, GZMH, GZMA, as well as co-stimulatory molecule CD2 (Supplementary Data 1C). On the other hand, CD8_2 were more enriched in tumor samples (Supplementary Fig. 9A) and uniquely expressed multiple heat-shock proteins (HSPA1B, HSPA6, HSPA1A), resembling a specific cluster of dysfunctional CD8^+^ T cells recently identified in melanoma^24^.

The activity and function of T cells are profoundly impacted by other stromal cells in the microenvironment, particularly myeloid cells, via extracellular signaling through ligand-receptor interactions^25^. To explore this type of cell-cell interaction between myeloid (DC, monocyte, macrophage) and T cell subtypes, we applied the CellPhoneDB method^26^ in ESCC tumor and nonmalignant samples. Notably, the overall number of interactions between myeloid and T cells was substantially higher in tumor than nonmalignant samples (Fig. 5F). Moreover, a number of tumor-specific interactions such as the CXCL9-CXCR3 immune activation axis^27^ between the macrophages and T cells were identified (Supplementary Data 2). In addition, we found that key immune-checkpoint inhibitors and their ligands such as PD1-PDL1 (CD274) and CTLA4-CD80/86 had higher interaction score in tumor than that in normal samples (Fig. 5G), in concordant with our above observation that both CD4 and CD8 T cells were more exhaustive in tumor samples.

### Myeloid cellular diversity and activation trajectory in ESCC

**Fig. 6:**
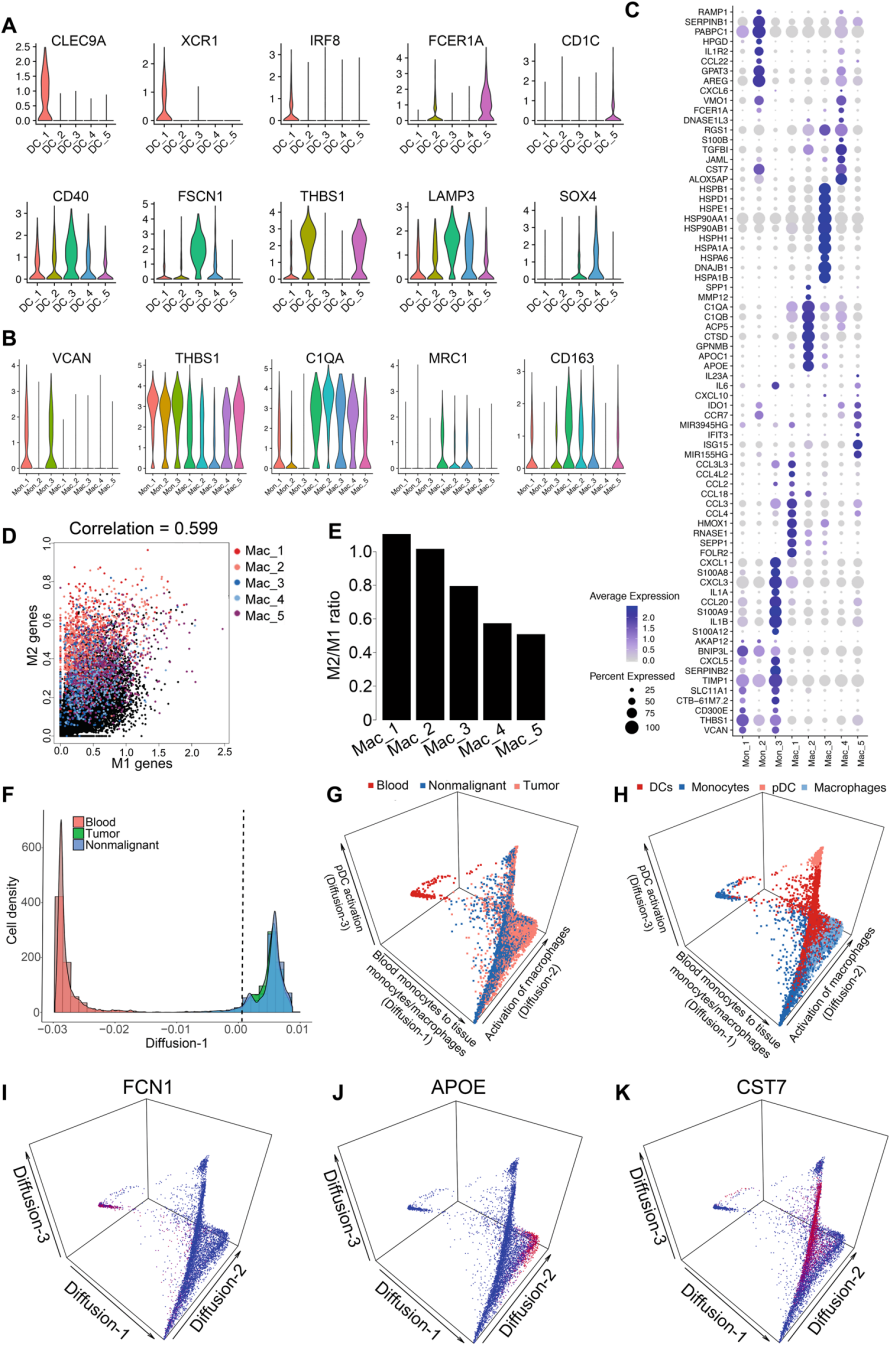
The cellular heterogeneity of myeloid compartment in ESCC. **A** Violin plots showing expression of representative markers in either DC (dendritic cell) or (**B**) monocyte and macrophage subsets. **C** Dotplot showing the expression of top 10 differentially expressed genes across monocyte and macrophage subsets. **D** Scatter plot showing the correlation of M1 and M2 gene signatures in individual macrophage subsets using all myeloid cells as background. **E** The expression ratio of M2 over M1 gene signatures. **F** Histogram of the distribution of myeloid cells along the Diffusion-1 component from either the PBMC (peripheral blood mononuclear cells), tumor or nonmalignant samples. **G** Diffusion component analysis of the myeloid compartment displayed in 3D plot showing the first 3 diffusion components colored by either sample origin or (**H**) annotated subsets. The 1st diffusion component reflected a trajectory from blood monocytes to tissue monocytes/macrophages, the 2nd diffusion component reflected the activation of monocyte-derived macrophages in tissue, and the 3rd diffusion component reflected a trajectory from pDCs (plasmacytoids dendritic cell) to more mature DC subsets. **I**–**K** 3D plots showing the correlation of representative genes with each diffusion component. Source data are provided as a Source Data file.

Recent single-cell profiling studies have revealed extensive heterogeneity in three major types of myeloid cells (DC, monocyte, macrophage) with distinct gene signatures observed across multiple cancer types^12,15,28^. Conventional DCs (cDC) are classified into two subsets, cDC1 and cDC2, and their primary function is to acquire tumor antigen, migrate into lymph nodes, and prime CD8^+^ and CD4^+^ T cells, respectively^29,30^. In the microenvironment of ESCC, DCs were notably diverse and clustered into five cDC subsets and a plasmacytoid (pDC) subset that expressed LILRA4 and IRF7 (Fig. 6A). Of these clusters, DC_1 expressed classic cDC1 signature genes including CLEC9A, XCR1, IRF8, BATF3, while DC_5 highly expressed conventional cDC2 markers, such as FCER1A, CD1C (Fig. 6A, Supplementary Data 1D). DC_3 was detected to have activated DC markers (CD40, FSCN1, CCR7), similar to a subset identified in lung tumors that lacks expression of key cDC1 and cDC2 genes^12,15,28^. DC_4 expressed myeloid precursor markers, such as the transcription factor SOX4 (Supplementary Data 1D), indicative of their immature feature. Given the high diversity of DC subsets in ESCC, further investigations are required to elucidate their functions in the ESCC microenvironment.

Across nonmalignant and tumor samples, we identified 3 monocyte and 5 macrophage clusters (Fig. 5A). Among the monocytic clusters, we found 2 classical CD14+ monocyte subsets, Mon_1 and Mon_3, which expressed conventional monocytic genes, such as VCAN, THBS1, and CD300E (Fig. 6B–C). Interestingly, both Mon_1 and Mon_3 also expressed the matrix metalloproteinase inhibitor TIMP1 and CXCL5 (the ligand for CXCR2) that promote recruitment of suppressive granulocytes and enhance tumor growth in mice^31^. In addition, Mon_3 highly expressed multiple cytokines (IL1A, IL1B, IL6)^32^ and chemokines (CXCL1^33,34^, CCL20^34^) known to suppress antitumoral immunity. Mon_3 also expressed exclusively S100A8/9, which were previously considered as monocyte markers in bulk RNA-Seq data. In line with our results, S100A8/9 expression was observed to be restricted in a subset of monocytes in lung cancer^28^. The other monocytic subset Mon_2 expressed SERPINB1 (Fig. 6C), a cytoplasmic serine protease inhibitor produced by neutrophils and monocytes to limit both inflammation and tissue damage^35^. Mon_2 also expressed CCL22, which has been shown to contribute to metastasis of head and neck tumors^36^. These expression signatures indicate immune-regulatory and pro-tumoral roles of different monocytic subsets in the ESCC microenvironment.

Among the five macrophage subsets in ESCC, Mac_1, Mac_2, and Mac_3 expressed higher anti-inflammatory “M2”-associated genes, including CCL18, MRC1, CD163, C1QA, APOE, SPP1, and TREM2 (Fig. 6B–E); most of these genes were also upregulated in tumors compared with nonmalignant samples (Supplementary Fig. 10A, Supplementary Data 1E). Nevertheless, these M2-like cells showed notable differences. Specifically, Mac_1 expressed multiple chemokines (CCL2, CCL3, CCL4) with established immunosuppressive roles. Mac_2 expressed Cathepsin genes (e.g., CSTA, CSTD) in a tumor-specific manner (Supplementary Fig. 10A), which are important for ECM remodeling. Indeed, Cathepsin-secreting macrophages have been characterized to promote tumor cell migration and invasion^37^. Mac_3 intriguingly expressed a number of nonclassical monocytic genes such as transcription factors KLF2/4 and NR4A1/2 (Supplementary Data 1E) as well as heat-shock proteins (Fig. 6C). Mac_5 was characterized by its specific expression of interferon-stimulated genes such as ISG15, OASL, IFIT2/3 (Fig. 6C). This suggests that Mac_5 may represent proinflammatory macrophages activated by interferon signaling. Indeed, Mac_5 expressed the lowest M2-like gene signature compared with other macrophage subsets (Fig. 6D–E). However, Mac_5 also expressed both IDO1 (Fig. 6C) and PD-L1 (Supplementary Fig. 10A), suggesting potential inhibitory activity against CD8^+^ T cells. In order to understand the gene expression programming in macrophages, we performed SCENIC analysis^38^ and identified 27 candidate “regulons” across different macrophage subsets (Supplementary Fig. 10B). Multiple candidates have been reported to play important roles in tumor-associated macrophages. For example, ATF3 has been identified as a regulator promoting the M2 suppressive phenotypes, and ATF3 empowers macrophages to enhance breast cancer metastasis^39^. Similarly, c-MYC is a key player in alternative macrophage activation in the tumor microenvironment, and contributes to tumor-promoting functions of tumor-associated macrophages^40,41^. These data together highlight the immune-suppressive functions by heterogeneous populations of macrophages in ESCC tumors.

To analyze further the activation and differentiation trajectories of immune cells in ESCC patients, we integrated a total of 20,924 PBMC cells from three matched ESCC patients (Supplementary Fig. 11A). In general, expected patterns of cellular states were observed comparing immune cells from different sample types: i) myeloid cells were more abundant in tissue samples, while B and T cells were more abundant in circulation (Supplementary Fig. 11A); ii) distinct separation of myeloid and T cell clusters between blood, nonmalignant and tumor samples, suggesting tissue-associated modulation of immune cells (Fig. 6F–G, Supplementary Fig. 11B). We then performed diffusion component analysis to identify gene expression patterns associated with the observed variation of cellular states. Focusing on the myeloid compartment, three major diffusion components were revealed (Fig. 6G–H). Correlation of the 2,000 most variable genes identified expression signatures associated with each diffusion component (Supplementary Fig. 11C, Supplementary Data 3). Specifically, the first component reflected a trajectory from blood monocytes to tissue monocytes/macrophages, congruent with a recent finding in breast cancer^12^. Circulation monocytic genes (FCN1, S100A4/8/9/12, CD52) were inversely correlated, while genes abundant in differentiated macrophages (such as HLA-DR genes and CD74) were positively correlated with the first diffusion component (Fig. 6H–I). CXCL8, a chemokine produced by activated macrophages, was also increased along with the first component (Supplementary Fig. 11C). The Diffusion-2 component was characterized by activated macrophage genes, such as APOE, C1Q(s), and IL1B (Fig. 6H, J), which likely reflected the activation of monocyte-derived macrophages in tissue, similar to the observed macrophage activation pathway in breast^12^ and head and neck cancer^15^. The third diffusion component was associated with DC-related markers FCER1G and FTL, and upregulated genes in differentiated DCs such as CST7 and DAPP1 (Fig. 6H, K), suggesting a trajectory from pDCs to more mature DC subsets in ESCC microenvironment. These analyses together suggest important transcriptional programs underlying distinct myeloid cellular trajectories and differentiation in ESCC microenvironment.

### Validation of candidate cell subsets by multiplexed IF staining

**Fig. 7:**
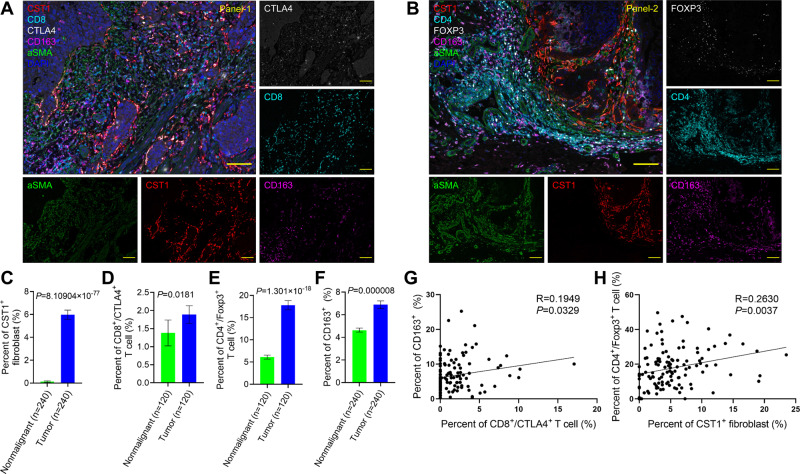
Validation of candidate cell subsets by multiplexed immunofluorescence (IF) staining. **A**–**B** Representative images of multiplexed IF staining of tumor samples using Panel-1 (**A**) and Panel-2 (**B**). Scale bar = 100 μm. **C–F** Quantification of CST1^+^ fibroblasts (**C**), exhaustive CD8^+^ T cells (**D**), Treg cells (**E**), M2-like macrophages (**F**) in nonmalignant (green) and tumor (blue) samples. The number of regions quantified are shown in the parentheses. Data are presented as mean values ± SEM. *P* values are calculated by two-tailed Mann Whitney *U* test. **G**–**H** Scatter plots showing the positive correlation (**G**) between exhaustive CD8^+^ T cells and M2-like macrophages (*n* = 120), and (**H**) between CST1^+^ fibroblasts and Treg cells (*n* = 120). Source data are provided as a Source Data file.

We next performed multiplexed 6-color immunofluorescence staining to extensively characterize the abundance and localization between the candidate immuno-suppressive immune cells (including macrophages and Tregs), CD8^+^ T cells as well as CST1^+^ fibroblasts. Specifically, we used the following two panels of combinations of antibodies for multiplexed IF assays: Panel-1 (Fig. 7A) consisted of αSMA + CST1 (for CST1^+^ fibroblasts), CD8 + CTLA4 (for exhaustive CD8^+^ T cells), CD163 (for M2-like macrophages); Panel-2 (Fig. 7B) consisted of αSMA + CST1, CD4 + FOXP3 (for Treg cells), CD163. Note that we chose αSMA instead of COL1A1 to further validate the presence of CST1^+^ fibroblasts.

These multiplexed IF staining were performed on 12 matched tumor and nonmalignant samples from ESCC patients, and an average of 10 regions were quantified for each sample. Using these data, we validated the low presence of CST1^+^ fibroblasts in nonmalignant samples (Fig. 7C), increased exhaustive CD8^+^ T cells (Fig. 7D), Treg cells (Fig. 7E), as well as M2-like macrophages (Fig. 7F) in tumor compared with nonmalignant samples. Moreover, the abundance of M2-like macrophages was modestly but significantly correlated with that of exhaustive CD8^+^ T cells in tumor samples (Fig. 7G), indicative of a negative regulation of CD8^+^ T cells by M2-like macrophages. In addition, we observed modest positive correlation between CST1^+^ fibroblasts and Treg cells in tumor samples (Fig. 7H). No correlation was noted between CST1^+^ fibroblasts with either exhaustive CD8^+^ T cells or M2-like macrophages, suggesting that CST1^+^ fibroblasts may play a more important role in regulating Treg cells in the ESCC microenvironment. We reason that this may be because CST1^+^ fibroblasts have the highest activity of TGF-beta signature. Indeed, it is well-established that TGF-beta can strongly promote the growth and activity of Treg cells in the tumor microenvironment^42^.

## Discussion

In this study, we unbiasedly reveal prominent heterogeneity in most of the cell types in ESCC stoma, particularly immune cells (myeloid and T cells) and fibroblasts. By both confirming recently identified subsets in other cancer types (e.g., Heat-shock protein-expressing CD8+ T cells, Cathepsin-secreting macrophages) and characterizing specific markers (e.g., CST1 for myofibroblast), this dataset represents a valuable resource for future investigation of cellular diversity of cancer ecosystem. We further identified many tumor-specific signals and pathways from ESCC stromal cells (e.g., inhibitory checkpoints in T cells and macrophages, EMT and TGF-beta pathways in myofibroblasts), suggesting cancer-specific regulation of the transcriptional programs of stromal cells.

Overall, analyses of both myeloid and lymphoid compartments highlight the immunosuppressive nature of the ESCC microenvironment: i) Both CD4^+^ and CD8^+^ T cell compartments express various immune checkpoints in a tumor-specific manner; ii) Treg cells are more enriched in tumor samples; iii) M2-like macrophages (Mac_1, Mac_2 and Mac_3) express anti-inflammatory factors, and even the M1-like subset (Mac_5) expresses PD-L1 specifically in tumor samples; iv) Different monocyte subsets express immune-regulatory and pro-tumoral factors.

A specific population of CST1^+^ myofibroblast is characterized by high activities in ECM remodeling, protein secretion, EMT, and TGF-beta pathways. In addition, this subset is fast-growing and most tumor-specific. CellPhoneDB analysis showed that the number of ligand-receptor interactions engaging CST1^+^ fibroblasts was generally much higher in tumor than in nonmalignant samples (Supplementary Data 4). Using orthogonal approaches including immunostaining, CST1^+^ myofibroblast is confirmed in ESCC tumor stromal, and exhibits prominent prognostic values. Moreover, CST1^+^ myofibroblast is observed in other types of cancers but not nonmalignant samples. In agreement with our data, higher CST1 expression was significantly associated with worse survival of colon cancer patients in TCGA bulk RNA-Seq data (Supplementary Fig. 7B). The *CST1* gene encodes Cystatin SN, a secretory protein, which inhibits cysteine proteinases. Previous studies on the function of CST1 in cancer cells reported that CST1 contributes to the cell proliferation, survival, and metastasis of multiple tumors types, including gastric^43^, breast^44^, and colon cancers^45,46^. However, in one study of ESCC samples^47^, the expression of CST1 in tumor cells was associated with better patient prognosis, which appears to be contradictory. Nevertheless, these previous works were all focused on the cancer-intrinsic function of CST1, and its biological role in fibroblast cells awaits further investigation.

Another notable fibroblast subset is ap-Fibro, which exclusively expresses MHC class II genes compared with other fibroblasts. Unlike CST1^+^ myofibroblast, ap-Fibro is observed in both the nonmalignant and cancer samples in ESCC. Consistently, MHC II expressing ap-Fibro was recently identified in both pancreatic cancer^16^ and normal pancreas^22^. In a PDAC mouse model, these MHC II expressing ap-Fibros were shown to actively present antigen to CD4^+^ T cells^16^. However, similar to the observation in PDAC, ap-Fibro in ESCC expressed negligible levels of the costimulatory genes (CD80 and CD86, Supplementary Fig. 12), suggesting that ap-Fibro has a different function in terms of antigen presentation compared with professional APCs.

In addition to the identification of fibroblast subsets, we also observed metabolic heterogeneity across different fibroblasts. For example, myofibroblasts show heightened oxidative phosphorylation, glycolysis, and cholesterol homeostasis pathways (Supplementary Fig. 6B), which is in line with their highly proliferative signature. On the other hand, F_4 has high scores of both fatty-acid metabolism and hypoxia, and weak oxidative phosphorylation activity (Supplementary Fig. 6B). Interestingly, F_4 also shows strong enrichment of GO terms “Response to unfolded protein” and “Regulation of cellular response to heat”, indicating that this subset might be under metabolic stress. In addition, gene regulatory analysis identified a total of 32 candidate regulons (Supplementary Fig. 13), including transcription factors with known functions in fibroblasts from different cancer types (e.g., TWIST1^48,49^, STAT1^50^, MYLK^51^, NFATC2^52^). Some of these factors had reported functions consistent with the gene signatures established by our scRNA-seq data. For example, TWIST1 is a recognized driver of cancer-associated myofibroblasts, and TWIST1-high myofibroblasts promote the proliferation, migration, and invasion of cancer cells^48,49^. Likewise, increased activity of STAT1 has been found in CAFs which enhance the proliferation and survival of cancer cells^50^. Concordantly, both the TWIST1 and STAT1 are highly and specifically enriched in the F_3 subset which we have annotated as pro-tumor myofibroblasts.

Finally, cancer-specific alterations of gene expression in ESCC stroma may suggest the design of immunotherapies. For example, tumor macrophages increase the expression of inhibitory checkpoint molecules while myofibroblasts upregulate oxidative phosphorylation and glycolysis pathways, which can be targeted using small-molecule inhibitors. Moreover, examination of CD8^+^ T cell supports current immune checkpoint blockade of PD-1 and CTLA-4, and further identifies additional inhibitory molecules as potential targets (e.g., LAG3, TIGIT). Therefore, distinctive biological characteristics of tumor stroma may indicate cancer-specific vulnerabilities and provide avenues for the development of innovative therapies.

## Methods

### Sample collection and single-cell isolation

Eleven patients who were pathologically diagnosed with ESCC were investigated in this study. None of the patients was treated with any antitumor therapy prior to tumor resection. The clinical characteristics of the patients are summarized in Supplementary Table 1. The adjacent nonmalignant esophageal tissues were obtained at least 5 cm away from the tumors. Peripheral blood mononuclear cells (PBMCs) were obtained from three patients prior to their surgical procedures. Clinical samples were collected from Cancer Hospital of Shantou University Medical College. This study was approved by the Ethics Committee of Shantou University Medical College. All patients provided written informed consent for sample collection and data analyses.

Clinical samples were collected immediately after surgery and were dissociated within 1 h using collagenase (17100017, Thermo Fisher Scientific). Briefly, tissues were minced into small pieces using a scalpel and transferred into a 15 mL tube containing HBSS (14025092, Thermo Fisher Scientific) and RPMI-1640 medium (1:1) supplemented with 200 U/mL collagenase, followed by a 4-hour incubation in a constant temperature oscillator at 37 °C, 110 rpm. After incubation, suspended cells were passed through a 40 μm cell strainers (352340, BD Falcon) and centrifuged at 300 g for 10 min. After washing twice with DPBS (14190144, Thermo Fisher Scientific), cell pellets were resuspended in DPBS buffer supplemented with 0.04% BSA. The entire mixed cell population was analyzed further without sorting or enriching for particular cell types.

PBMCs were isolated using Ficoll-Paque PLUS solution (17-1440-03, GE Healthcare) according to the manufacturer’s instructions. Briefly, 5 mL of fresh peripheral blood was collected prior to surgery in an EDTA anticoagulant tube and subsequently layered onto Ficoll-Paque PLUS. After centrifugation, lymphocytic cells at the plasma-Ficoll-Paque PLUS interface were carefully transferred to a new tube and washed twice with DPBS. Lymphocytic cells were resuspended with sorting buffer.

### Library preparation and sequencing

The scRNA-Seq libraries were prepared from individual cells using the 10X Genomics platform. The Chromium Single Cell 3’ Library & Gel Bead Kit v2 (PN-120237), Chromium Single Cell 3’ Chip kit v2 (PN-120236) and Chromium i7 Multiplex Kit (PN-120262) were used according to the manufacturer’s instructions. Briefly, for each sample, approximately 9000 cells were loaded onto the 10X Genomics Chromium Controller machine for Gel Beads-in-Emulsion (GEM) generation. Reverse transcription was performed using a C1000 Touch Thermal Cycler with a Deep Well Reaction Module (Bio-Rad) using the following program: 55 °C for 2 h; 85 °C for 5 min; hold 4 °C. cDNA was recovered, purified, and amplified to generate sufficient quantities for library preparation. All single-cell libraries were sequenced on the Illumina Hiseq X (PE150).

### scRNA-Seq analysis

#### Preprocessing, QC, and data integration

Raw reads were aligned to the hg38 reference genome, UMI (unique molecular identifier) counting was performed using Cell Ranger v.2.1.1 (10X Genomics) pipeline with default parameters. This yielded an average of 7523 / 2581 / 1648 cells (1080/1663/2151 genes per cell) for PBMC, nonmalignant, and tumor samples, respectively. Potential doublets were detected and filtered using DoubletFinder based on the expression proximity of each cell to artificial doublets^53^. After removing cells with high mitochondrial content (>= 20%), a total of 21,355 nonmalignant, 19,882 tumor and 20,924 PBMC cells were retained for downstream analysis.

We used Seurat v3 anchoring integration method^18^ based on canonical correlation (CC) analysis. By default, 2000 highly variable genes were used for finding alignment anchors and 30 CC dimensions were used for defining neighbor search space, with the function *FindIntegrationAnchors*. The anchors, determined and scored for all sample pairs, were then integrated together to assemble the reference to account for batch effect and sample variations using the function *IntegrateData* in the Seurat package. No batch effect was observed in the integrated data (Supplementary Fig. 1). We integrated 9 nonmalignant and 13 tumor samples and determined the major cell types based on well-defined markers (described in the next paragraph).

#### High-dimensional reduction and clustering analysis

Principal Component Analysis (PCA) was used (Seurat package) with the number of optimal PCA dimensions being defined using standard deviations saturation plot for further non-linear high-dimensional reduction method. We used UMAP (Uniform Manifold Approximation and Projection)^54^ for visualization of cell types and clusters. Clustering was performed for integrated expression values based on shared-nearest-neighbor (SNN) graph clustering (Louvain community detection-based method) using *FindClusters* in Seurat package. Robustness and further clustering analysis were evaluated by clustree method at different resolutions from 0.1 to 3^55^ with different representative markers. Specifically, the following marker genes were used for global annotation of cell types: Epithelial/Tumor: EPCAM, Keratin genes (KRT7, KRT8, KRT17), SPRR3; T cells: CD3E, CD3D, TRBC1/2, TRAC; Myeloid cells: LYZ, CD86, CD68, FCGR3A; B cells and plasma cells: CD79A/B, JCHAIN, IGKC, IGHG3; Endothelial cells: CLDN5, FLT1, CDH1, RAMP2; Fibroblasts: DCN, C1R, COL1A1, ACTA2; Smooth muscle cells: TAGLN, CNN1; Mast cells: TPSAB1. First, we used the clustering results at resolution 3 for all 44,085 cells from both nonmalignant and tumor samples to determine the major cell types based on the above markers for 53 clusters (Supplementary Fig. 2). Cell type scores, as the average expression of those known markers, were assigned for each of 53 clusters in order to determine the initial call of major cell types. Further sub-clustering was performed for immune cells and fibroblasts for in-depth investigation and better annotation of clusters based on more specific markers within each cell type. Any cellular subset was required to account for at least 2% of total cells from at least 2 different samples. The PBMC data was separately analyzed and annotated using the same computational framework, and was then integrated together with immune cells extracted from the nonmalignant and tumor samples.

### Differential expression analysis and gene set enrichment analysis (GSEA)

We used *FindAllMarkers and FindMarkers* in *Seurat* package with MAST differentially expression analysis method^56,57^, one of the leading methods in the benchmark study, to find gene signatures of one subset versus the rest and to identify the differentially expressed genes between tumor and nonmalignant samples within each subset, respectively. It was run with cutoff logfc 0.25 of a subset compared to the rest (>=15% of cells in the corresponding subset were required to have expression of the candidate gene).

We used Gene Set Enrichment Analysis (GSEA)^58^ and top GO^59^ to determine the enrichment of cancer hallmark (H dataset) and Biological Process Gene Ontology (GO, C5 dataset) terms, respectively. Linear regression was used to compute the enrichment of a specific subset compared with the rest in each cellular compartment, similar to the method described previously^13^.

### Cell-cell communication analysis using CellPhoneDB

We inferred cell-cell interactions based on ligands (originating from myeloid cell types) and receptors (in the T cell subsets) using permutation test as described in CellPhoneDB^26^. Statistically significant interacting pairs were identified with adjusted *P* values < 0.05. We used the *signiciant_mean* value output from CellPhoneDB as the interaction score for each ligand-receptor pair to represent the total mean of average expression values of the individual partner in each interacting pair. We merged all the myeloid subsets into 3 major myeloid cell types (macrophages, monocytes, dendritic cells) and all the T cell subsets into 3 major T cell types (CD4, CD8 and Treg) to achieve sufficient statistical power.

### Diffusion component analysis

Diffusion component analysis^60^ was performed to identify the components representing gene expression variations across different cellular compartments. We performed destiny R package^61^ on myeloid cells across all tumor, nonmalignant and PBMC samples based on the top 2000 most variable genes identified using the regression method from *FindVariableFeatures* (vst parameter) in *Seurat* package. The first three diffusion components were visualized using *plot3D* R package. Spearman rank correlation analysis was performed to identify both the positively and negatively correlated genes.

### Immunohistochemistry (IHC) staining

Formalin-fixed, paraffin-embedded ESCC specimens were obtained from Shantou Central Hospital. All the specimens were pathologically confirmed as ESCC and the clinical staging of the tumors were classified according to the seventh edition of the tumor-node metastasis (TNM) system of the American Joint Committee on Cancer^62^. Clinicpathological characteristics of patients are summarized in Supplementary Table 2. Ethical approval was obtained from the ethical committee of the Medical College of Shantou University.

IHC staining was performed as described previously^63^. Briefly, 4 μm thick sections were dewaxed in xylene, rehydrated in alcohol, and incubated in 3% hydrogen peroxide for 10 min to block endogenous peroxidase activity. Slides were incubated with 10% normal goat serum in PBS for 10 min at room temperature to block nonspecific binding. Then slides were incubated overnight at 4 °C with the primary antibody for CST1 (1:400, 16025-1-AP, ProteinTech). After rinsing with PBS, slides were incubated with PV-9000 2-step Polymer Detection System (PV-9000, ZSGB-BIO) and the primary antibody was detected with Liquid DAB Substrate Kit (ZLI-9018, ZSGB-BIO). Finally, slides were counterstained with hematoxylin, dehydrated, and mounted.

### Automated image analysis and scoring

An automated quantitative pathology imaging system (Perkin Elmer)^63^ was applied. Briefly, we used Vectra 2.0.8 for automated image acquisition, and obtained 20–40 images from the area containing the expression of CST1 of the whole slide at 20X magnification. Spectral libraries were constructed by Nuance 3.0 software and then loaded into InForm 1.2 advanced image analysis software to separate positive from negative cells with a single threshold. It provided a score showing the number of cells with positive CST1 expression of each high-power image. The mean value of the scores of each case was determined. For statistical analysis, the protein expression score was divided into two subgroups, high-expression and low-expression, on the basis of X-tile software analysis^64^.

### Immunofluorescence staining

Four μm thick sections were dewaxed in xylene, rehydrated in alcohol, followed by heat-induced antigen retrieval in 0.1 M 95–99 °C sodium citrate (pH 6.0) for 10 min. After washing, sections were incubated with 5% donkey serum in PBS for 60 min at room temperature to block nonspecific binding. Subsequently, slides were incubated overnight at 4 °C with the first primary antibody, and then incubated at room temperature for 60 min in a dark chamber with the first fluorophore-conjugated secondary antibody diluted in PBS with 5% donkey serum. Slides were washed with PBS and then incubated with the second primary antibody, followed by staining with the second fluorophore-conjugated secondary antibody diluted in PBS with 5% donkey serum. Both of the last two steps were incubated at room temperature for 60 min in a dark chamber. The following antibodies were used: polyclonal rabbit antihuman CST1 (1:400, 16025-1-AP, ProteinTech), Alexa Fluor 647-conjugated Affinipure donkey antirabbit (1:1000, 711-605-152, Jackson ImmunoResearch), polyclonal rabbit antihuman COL1A1 (1:100, ab34710, Abcam), Alexa Fluor 488-conjugated Affinipure donkey antirabbit (1:1000, 711-545-152, Jackson ImmunoResearch) and monoclonal mouse antihuman HLA-DR (ready to use (no dilutions), ZM-0136, ZSGB-BIO), Alexa Fluor 647-conjugated Affinipure donkey antimouse (1:1000, 715-605-150, Jackson ImmunoResearch), monoclonal rabbit antihuman VIM (ready to use (no dilutions), ZA-0511, ZSGB-BIO), Alexa Fluor 488-conjugated Affinipure donkey antirabbit (1:1000, 711-545-152, Jackson ImmunoResearch). Finally, slides were counterstained with 4′,6-diamidino-2-phenylindole (DAPI) (1:2000, D9564-10MG, Sigma Aldrich), and mounted.

We used Vectra 2.0.8 for automated image acquisition, and obtained randomly 30 images from the whole slide at 20X magnification. InForm 1.2 advanced image analysis software was used to separate positive from negative cells with a single threshold. The average value of the score of each case was calculated.

### Multiplexed immunofluorescence staining

Multiplexed immunofluorescence staining of 4 μm formalin-fixed, paraffin-embedded sections was performed using the PANO 7-plex IHC kit (cat 0004100100, Panovue, Beijing, China) and the PANO 4-plex IHC kit (cat 0001100100, Panovue, Beijing, China) according to the manufacturer’s instruction. Different primary antibodies were sequentially applied, followed by horseradish peroxidase-conjugated secondary antibody incubation and tyramide signal amplification. Glass slides were microwave heat-treated following each round of TyramideSignal Amplification. Nuclei were stained with 4′-6′-diamidino-2-phenylindole (DAPI, D9542, Sigma-Aldrich) after all the human antigens were labeled.

The following antibodies were used: CST1 (1:300, 16025-1-AP, ProteinTech), CTLA4 (1:100, ab237712, Abcam), HLA-DR (1:200, ab92511, Abcam), CD45 (1:300, BX00087, Biolynx), VIM (1:300, #5741, CST), CD163 (1:300, #93498, CST), CD8A (1:300, #70306, CST), aSMA (1:50, ab7817, Abcam), FOXP3 (1:50, BLG320202, Biolegend), CD4 (1:200, BX22300130, Biolynx).

To obtain multispectral images, stained slides were scanned using the Polaris System (PerkinElmer, Massachusetts, USA), which captures the fluorescent spectra from 420 to 720 nm with identical exposure time. Images were analyzed using Inform advanced image analysis software (PerkinElmer, Massachusetts, USA). A spectral library and spectral unmixing algorithm were created by using unstained and single Opal dye-stained images. Using this spectral library, we reconstructed images and extracted targeted cells for statistical analyses. 10-15 random high-power fields (20X magnification) inside the region of interest were analyzed per sample.

### Statistical analysis

Statistical analyses were performed using SPSS 19.0 software (IBM). Survival analysis was performed by the Kaplan-Meier method with the log-rank test. Univariate and multivariate analyses were performed based on the Cox proportional hazards regression model. Associations of CST1 expression with clinicopathological characteristics were determined by Fisher’s Exact Test. Differences with a 2-tailed *P* value less than 0.05 were considered statistically significant.

### Reporting summary

Further information on research design is available in the Nature Research Reporting Summary linked to this article.

## Supplementary information

Supplementary informations

Description of Additional Supplementary Files

Dataset 1

Dataset 2

Dataset 3

Dataset 4

Reporting Summary

## Data Availability

The sequencing data of scRNA-seq of this study was deposited into National Center for Biotechnology Information Sequence Read Archive (SRA) under accession number PRJNA777911. Public microarray RNA expression data of ESCC were retrieved from GEO database (GSE53624). Public scRNA-seq data from lung cancer, colon cancer and HNSCC were downloaded from E-MTAB-6149 (lung cancer), E-MTAB-6653 (lung cancer), GSE81861 (colon cancer) and GSE103322 (HNSCC), respectively. Source data are provided with this paper.

## Source data

Source data
