# Supplementary material for: Integrated single-cell transcriptome analysis reveals heterogeneity of esophageal squamous cell carcinoma microenvironment
Source: Nat Commun. 2021 Dec 17;12:7335. doi: 10.1038/s41467-021-27599-5 (PMC8683407; doi:10.1038/s41467-021-27599-5)
Supplement: Supplementary file 1 — Supplementary informations [file 41467_2021_27599_MOESM1_ESM.pdf]

Supplementary Figure 1

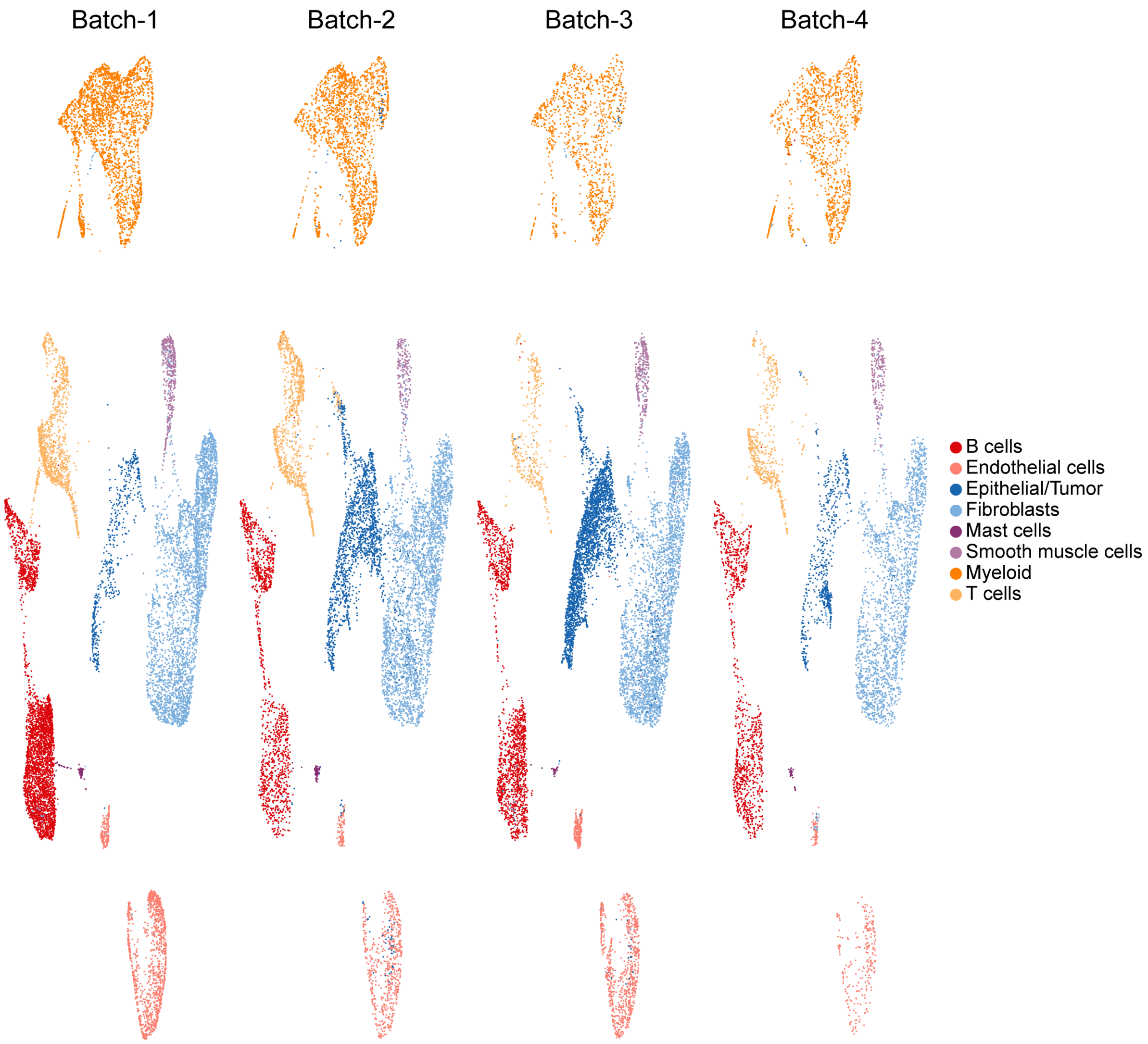

Supplementary Figure 1. UMAP plots of annotated major cell types from 4 sequencing batches.

Supplementary Figure 2

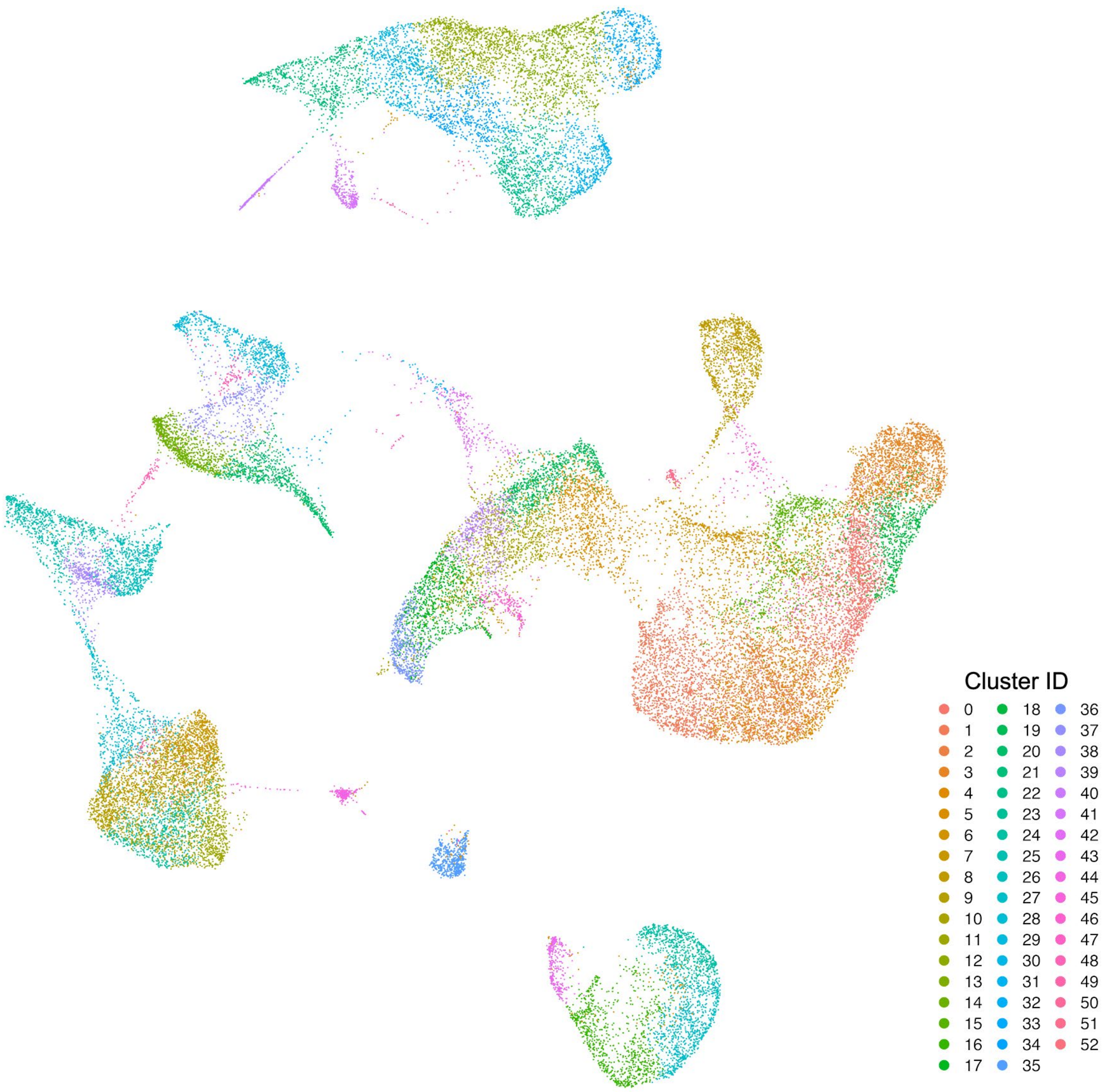

Supplementary Figure 2. UMAP plot of all 53 clusters at Seurat resolution 3.0 which were used for the assignment of major cell types based on well-defined markers.

Supplementary Figure 3

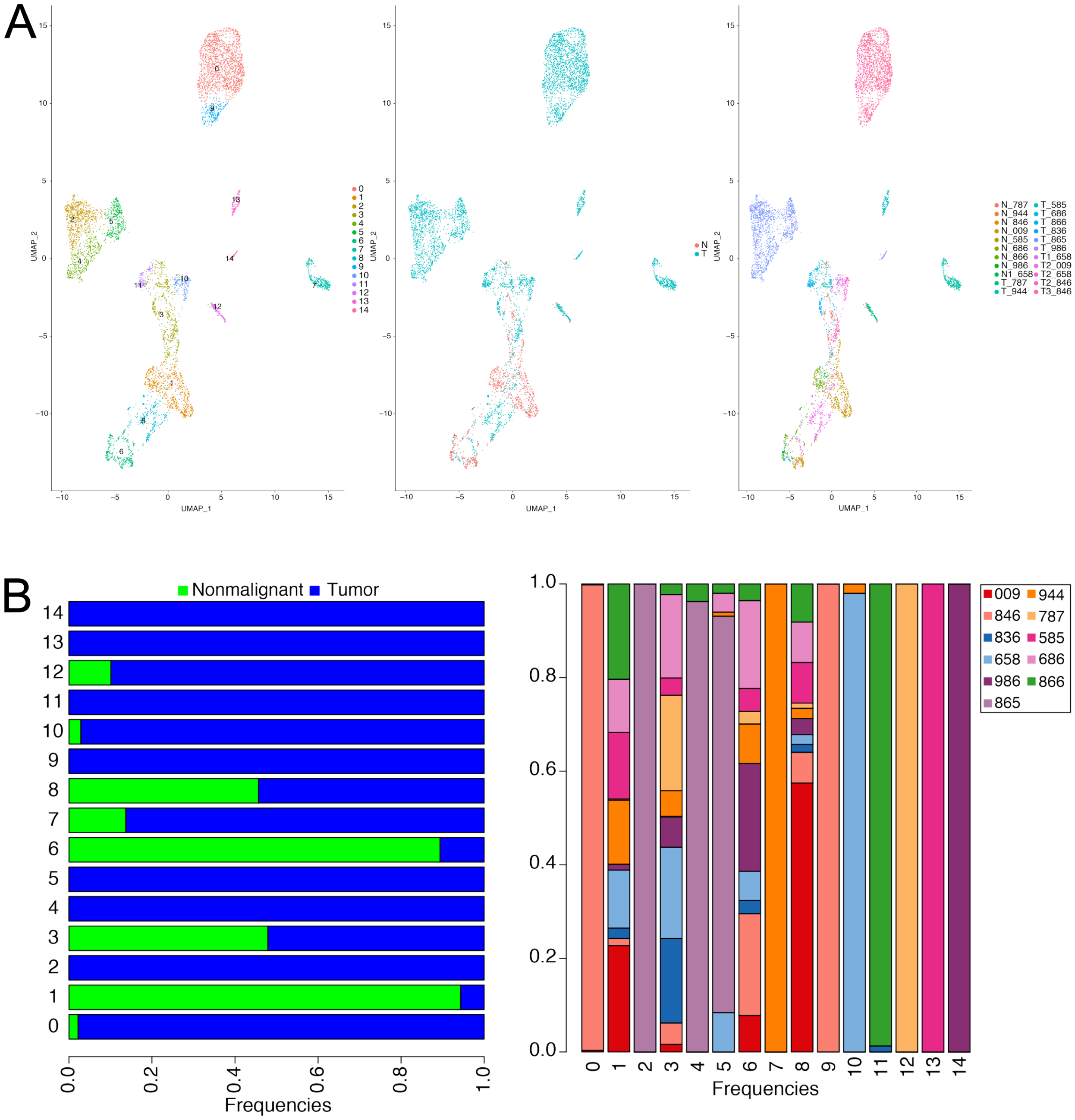

Supplementary Figure 3. (A) UMAP visualization of the clustering of epithelial/tumor cells from all nonmalignant and tumor samples, color coded by either major cell type (left), sample type (middle) or patient origin (right). **(B)** The frequency of each cluster in nonmalignant and tumor samples (left), and in each of the 11 patients (right). Source data are provided as a Source Data file.

Supplementary Figure 4

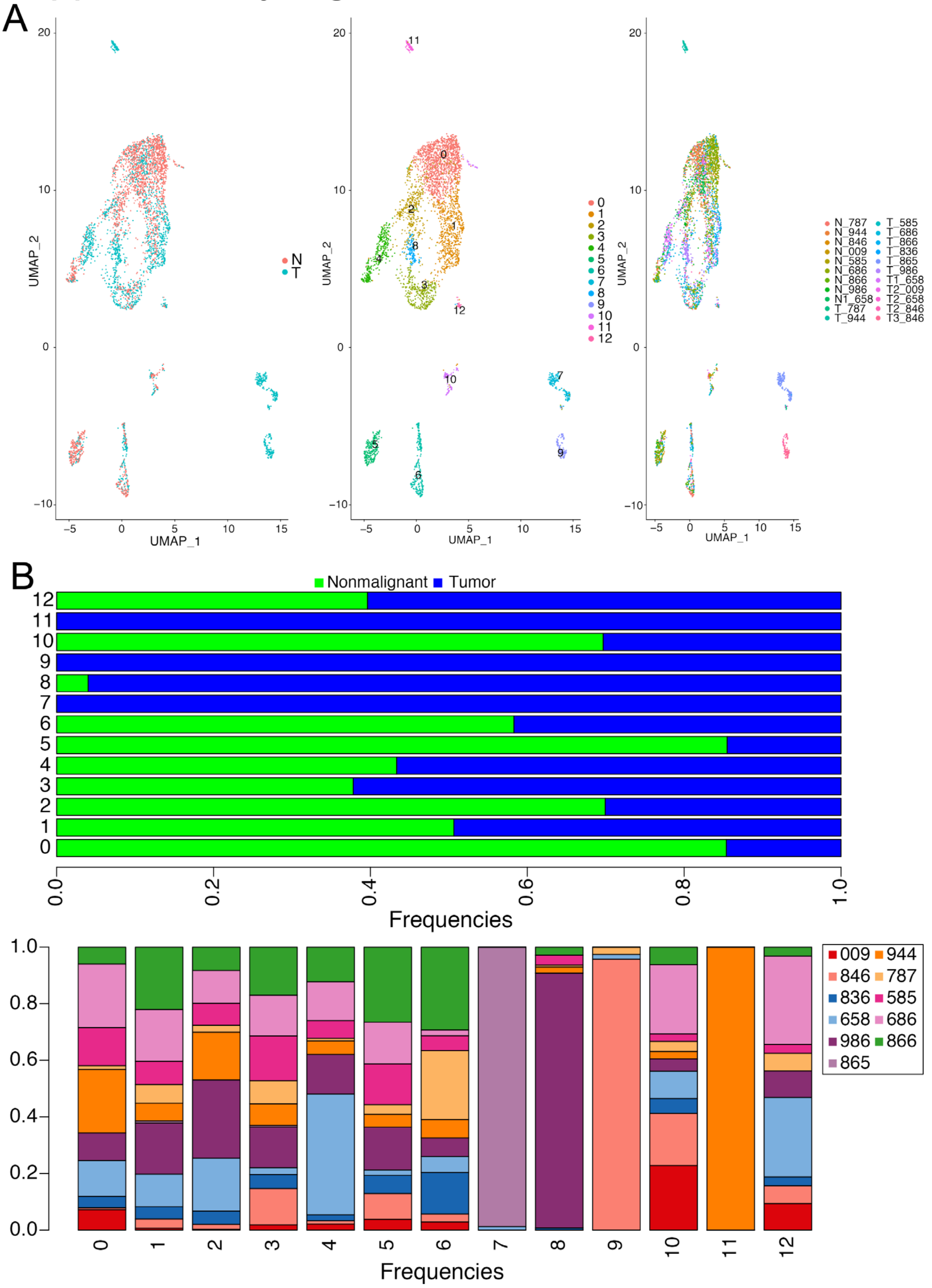

Supplementary Figure 4. (A) UMAP visualization of the clustering of all T cells from all nonmalignant and tumor samples, color coded by either sample type (left), major cell type (middle) or patient origin (right). **(B)** The frequency of each cluster in nonmalignant and tumor samples (upper), and in each of the 11 patients (lower). Source data are provided as a Source Data file.

Supplementary Figure 5

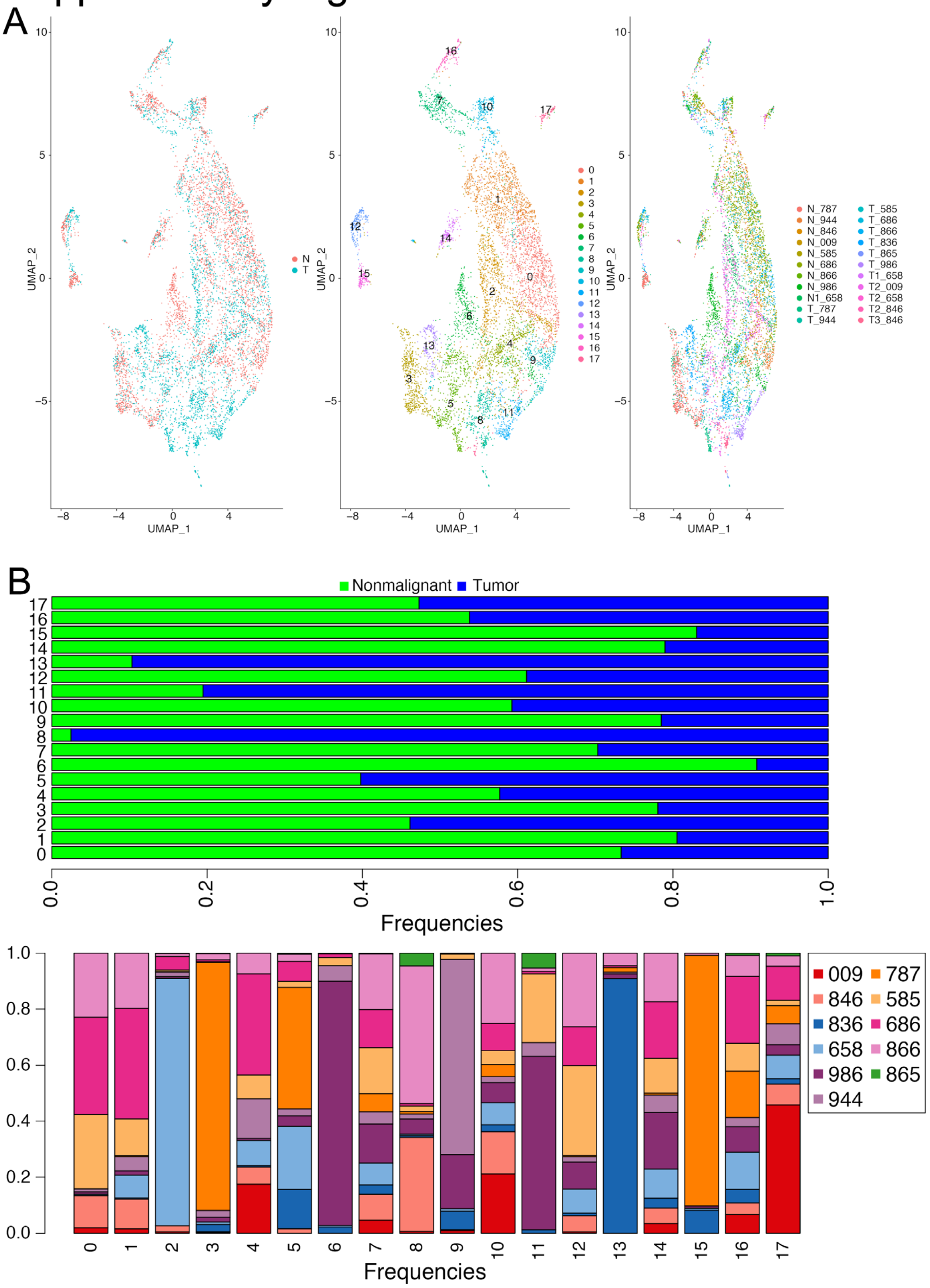

Supplementary Figure 5. (A) UMAP visualization of the clustering of all myeloid cells from all nonmalignant and tumor samples, color coded by either sample type (left), major cell type (middle) or patient origin (right). **(B)** The frequency of each cluster in nonmalignant and tumor samples (upper), and in each of the 11 patients (lower). Source data are provided as a Source Data file.

Supplementary Figure 6

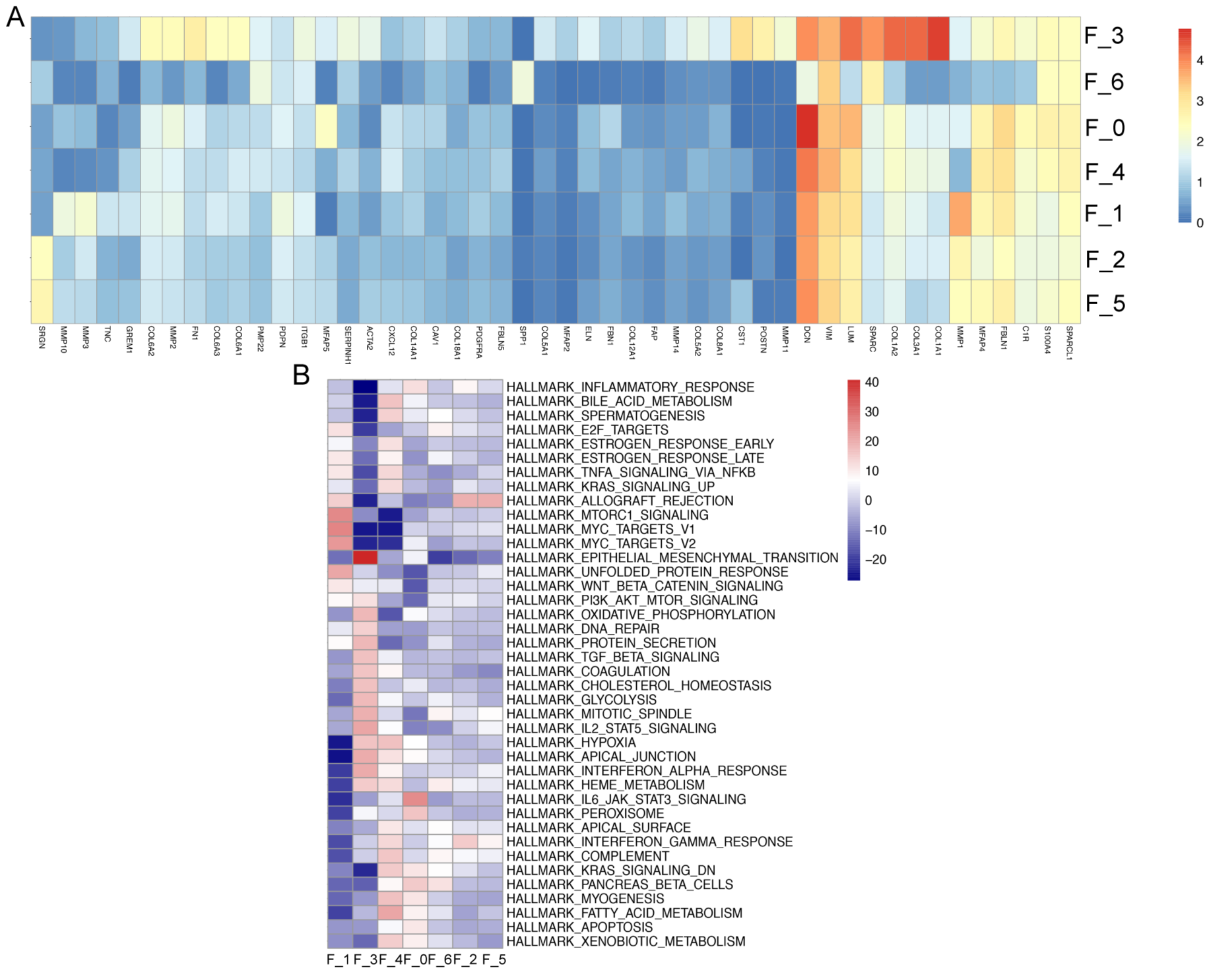

Supplementary Figure 6. (A) Heatmap showing the expression of ECM genes and fibroblast marker genes across all fibroblast subsets. (B) Heatmap of t values from hallmark gene set enrichment analysis for each fibroblast subset. Source data are provided as a Source Data file.

Supplementary Figure 7

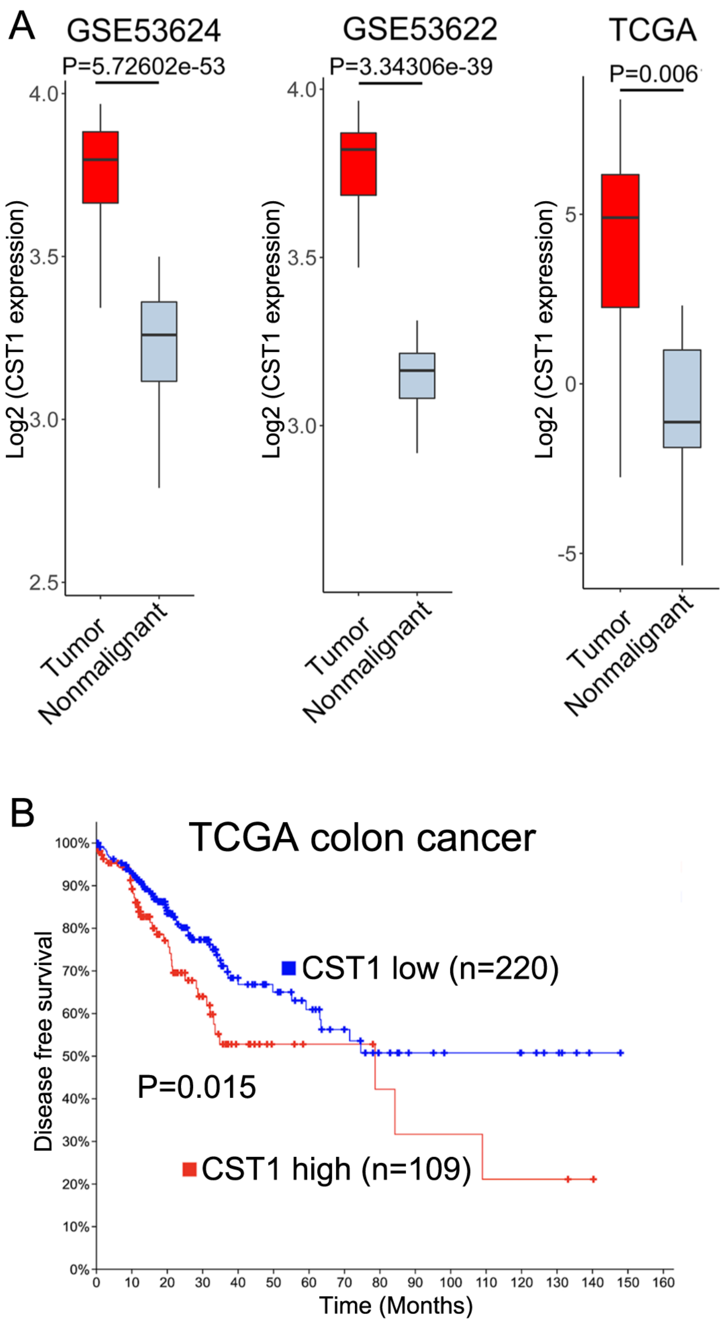

Supplementary Figure 7. (A) Validation of CST1 expression using bulk mRNA expression datasets of three different ESCC cohorts. n = 60 paired tumor and nonmalignant samples in GSE53622, n = 119 paired tumor and nonmalignant samples in GSE53624, n = 80 tumor and 11 nonmalignant samples in TCGA. The middle bar represents the median, and the box represents the interquartile range; whiskers indicate the maximum and minimum values. P values are calculated by two-tailed T test. (B) Kaplan-Meier curve of disease-free survival of colon cancer patients stratified by the average of CST1 mRNA expression in TCGA cohort. P values are calculated by Logrank test.

Supplementary Figure 8

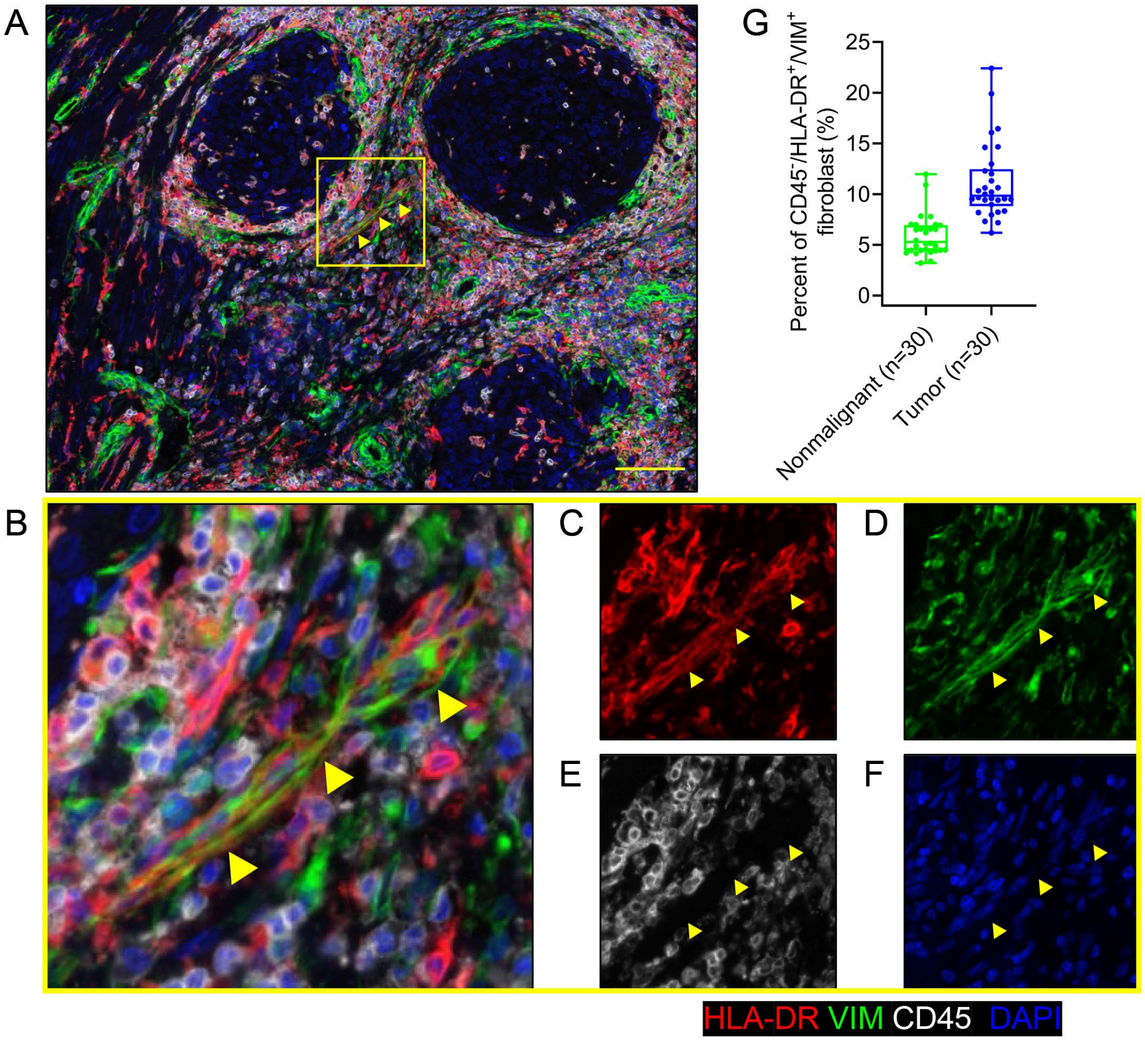

Supplementary Figure 8. (A) A representative image of multiplexed IF staining of a tumor sample. (B) A digital zoom in of the yellow square from (A), with yellow arrowheads highlighting CD45$^-$ HLA-DR$^+$ VIM$^+$ cells. (C-F) showing individual staining signals of HLA-DR (C), Vimentin (D), CD45 (E) and DAPI (F). (G) Quantification of the percentage of CD45$^-$ HLA-DR$^+$ VIM$^+$ in tumor and nonmalignant samples. Scale bar = 100 μm. The middle bar represents the median, and the box represents the interquartile range; whisker indicates the maximum value and minimum value. Source data are provided as a Source Data file.

Supplementary Figure 9

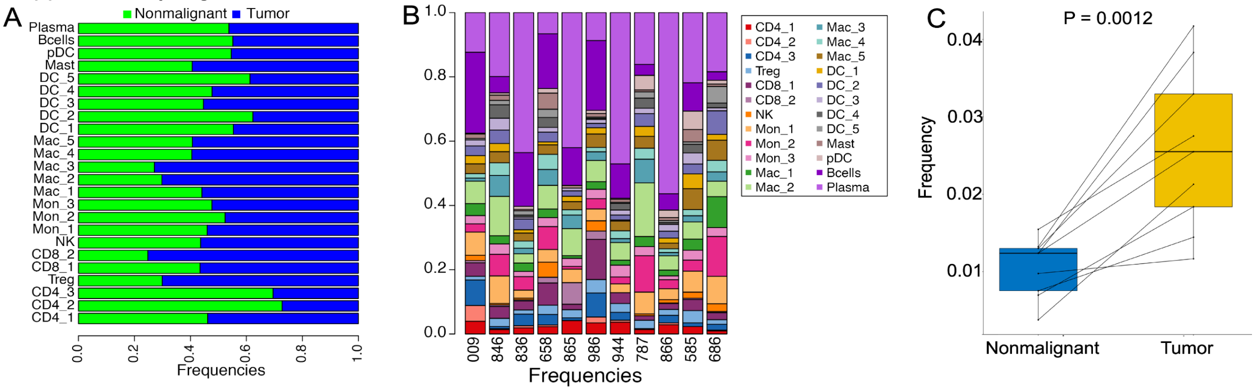

Supplementary Figure 9. (A) The fraction of each of the 24 immune cell types in nonmalignant and tumor samples, and (B) in each of the 11 patients (an analysis restricted within tumor samples). (C) Box plots showing the pairwise analysis of relative proportions of Treg cells in tumor versus nonmalignant samples. *n* = 9 for both nonmalignant and tumor samples. The middle bar represents the median, and the box represents the interquartile range; whiskers indicate the maximum and minimum values. *P* values are calculated by Wilcox test. Source data are provided as a Source Data file.

Supplementary Figure 10

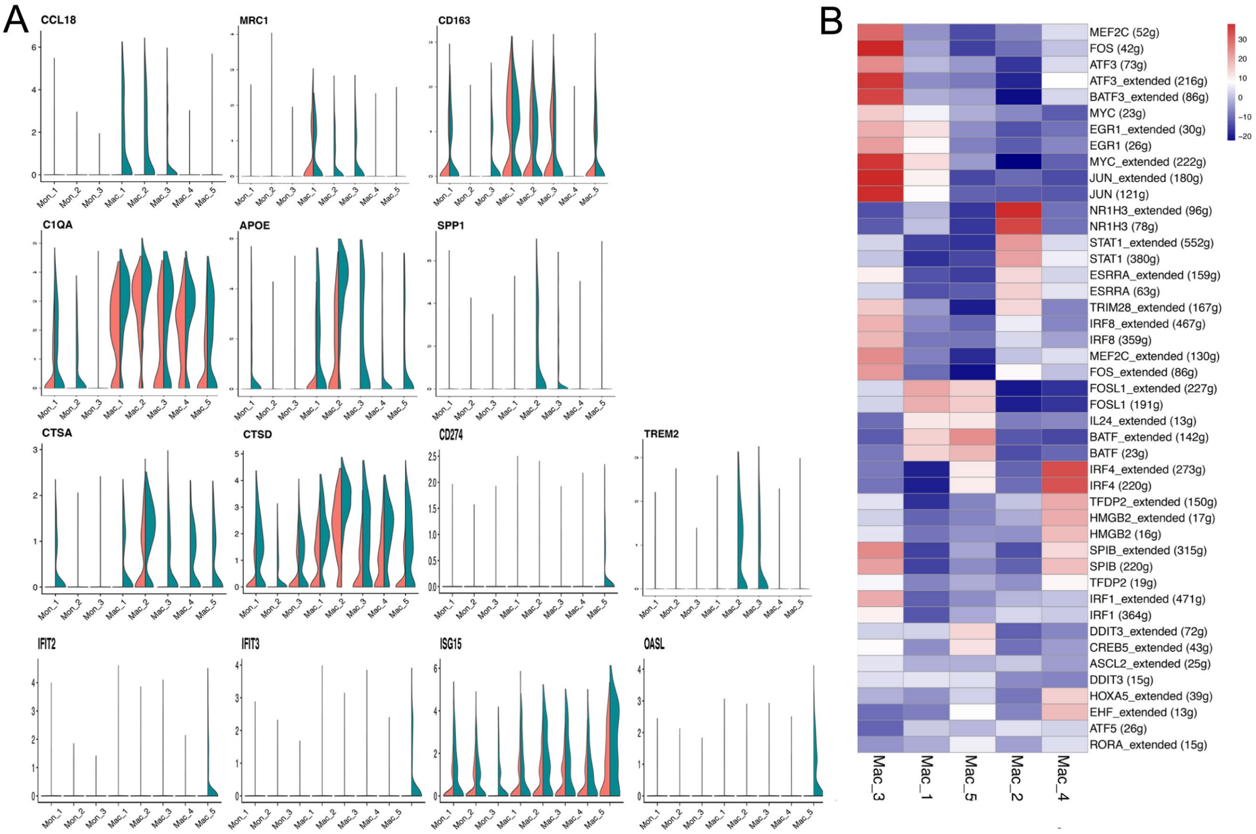

Supplementary Figure 10. (A) Violin plots of normalized expression levels of representative genes across monocyte and macrophage subsets, comparing tumor (blue) and nonmalignant (pink) samples. (B) Heatmap of t values from SCENIC analysis using linear model testing regulon activity for each subset from macrophages. Source data are provided as a Source Data file.

Supplementary Figure 11

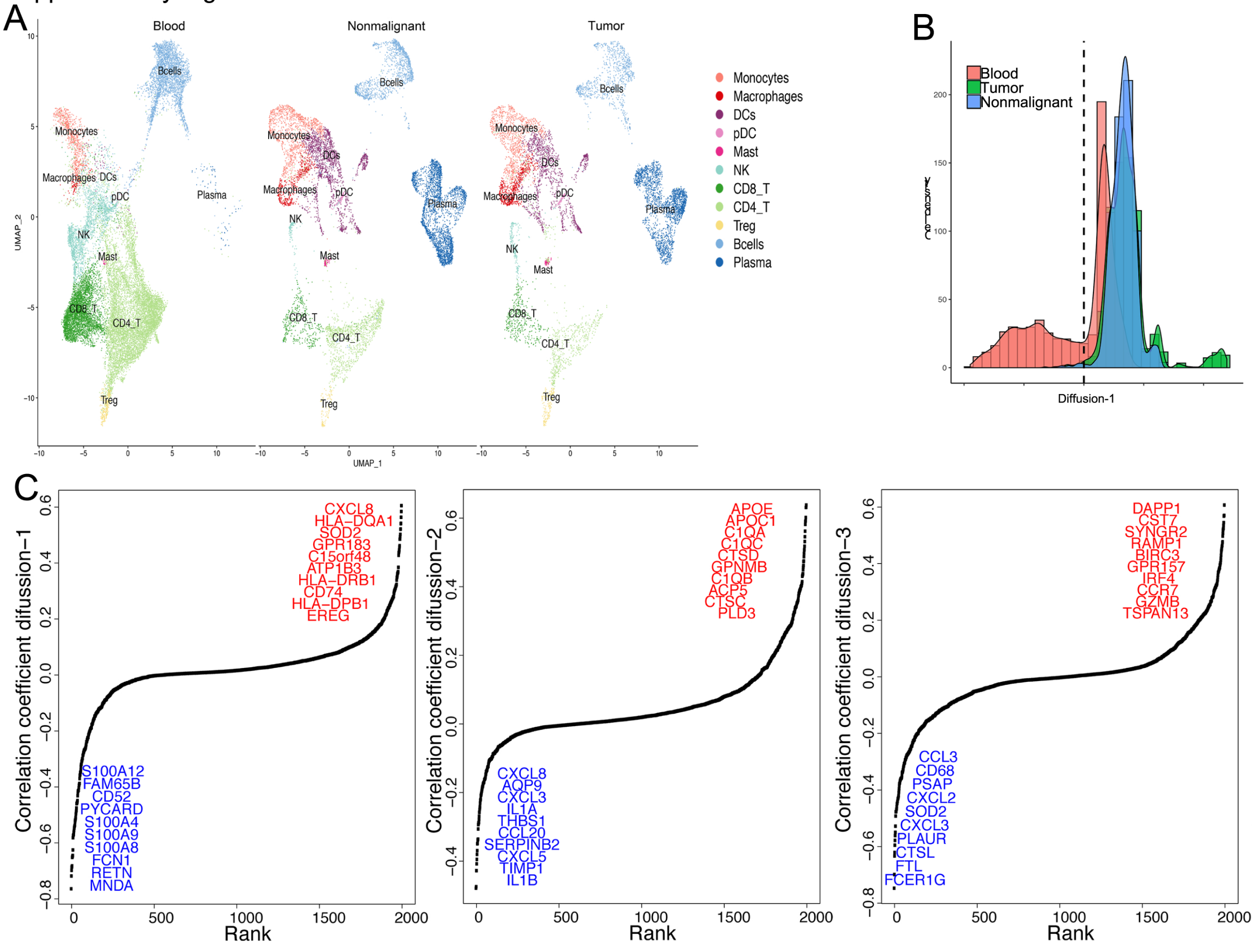

Supplementary Figure 11. (A) UMAP plots showing the integration of all immune cells extracted from the PBMC, tumor and nonmalignant samples. (B) Histogram of the distribution of T cells along the Diffusion-1 component from the PBMC, tumor and nonmalignant samples. (C) Correlation analysis of the 2,000 most variable genes with each of the three diffusion components ranked by correlation coefficient. Top 10 most positively correlated (red) and negatively correlated (blue) and genes are highlighted.

Supplementary Figure 12

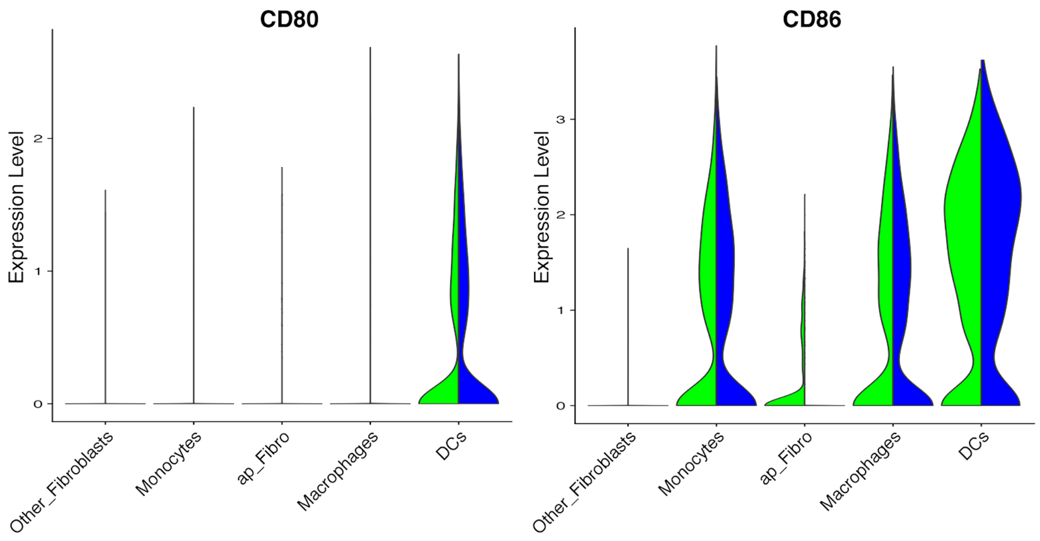

Supplementary Figure 12. Violin plots of normalized expression levels of CD80 and CD86 across different fibroblast and myeloid cell types, comparing tumor (blue) and nonmalignant (green) samples.

Supplementary Figure 13

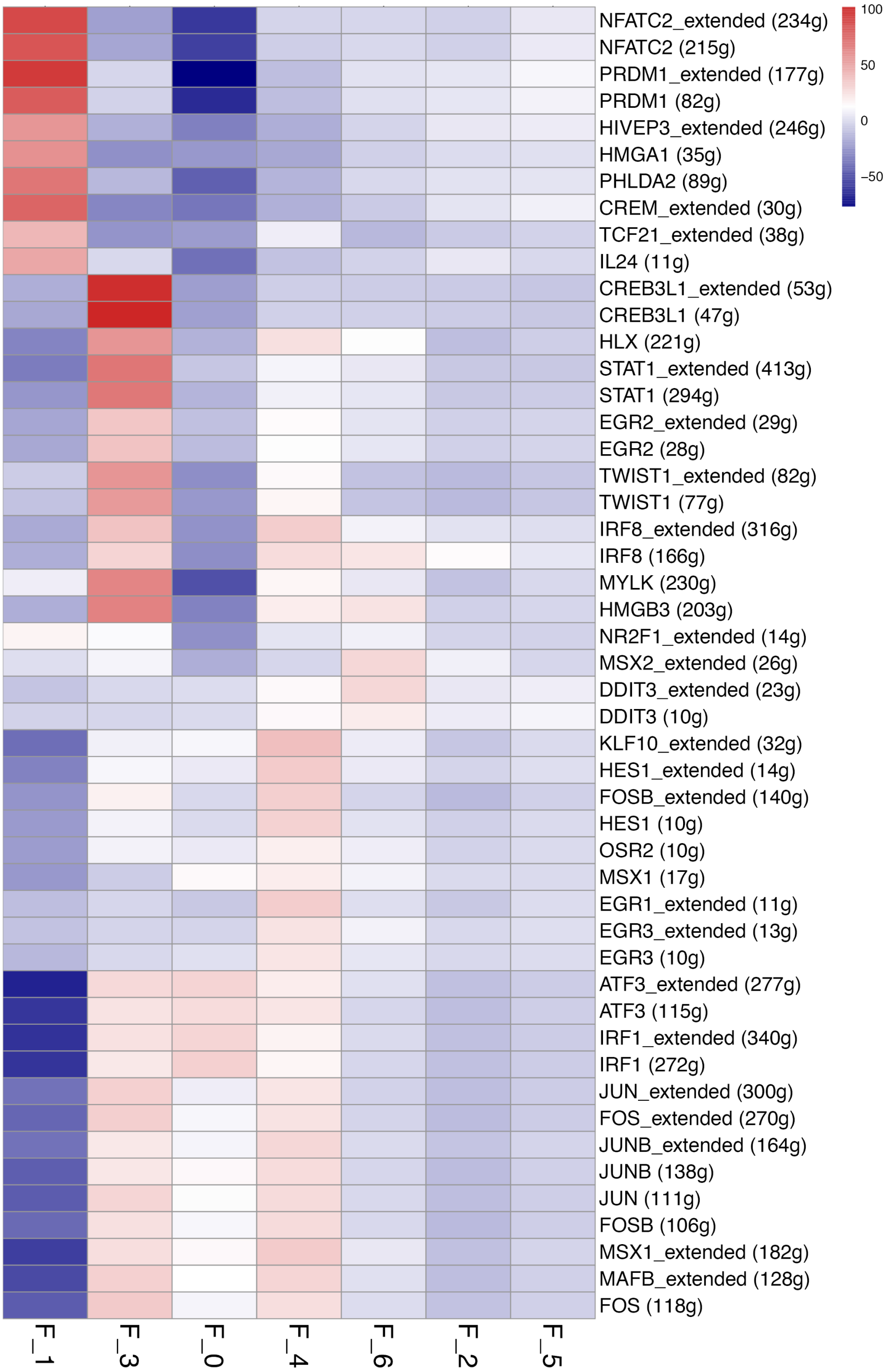

Supplementary Figure 13. Heatmap of t values from SCENIC analysis using linear model testing regulon activity for each subset from fibroblasts. Source data are provided as a Source Data file.

Supplementary Table 1. The clinicopathological parameters of 11 patients profiled by scRNA-seq

Patient ID	Histology	Stage	Sample type
787	squamous cell carcinoma	IIb	N/T
865	squamous cell carcinoma	IIIc	T
944	squamous cell carcinoma	IIIc	N/T
986	squamous cell carcinoma	Ib	N/T
658	squamous cell carcinoma	IIb	N/T1/T2
846	squamous cell carcinoma	IIIa	N/T2/T3
009	squamous cell carcinoma	0	N/T2
836	squamous cell carcinoma	Ia	T
686	squamous cell carcinoma	IIb	B/N/T
866	squamous cell carcinoma	IIIc	B/N/T
585	squamous cell carcinoma	IIIa	B/N/T

Abbreviations: B, PBMC; N, nonmalignant; T, tumor

Supplementary Table 2. The clinicopathological characteristics of the IHC cohort of ESCC patients

Clinical and pathological indexes	Case No.	5-year OS (%)	*P**	5-year DFS (%)	*P**
Specimens	154				
Mean age	58.51				
Age (year)					
≤58	81	57.6	0.398	47.5	0.406
>58	73	44.8		32.8	
Gender					
Male	115	48.8	0.157	36.2	0.069
Female	39	59.5		54.2	
Tumor size					
≤3cm	55	56.5	0.885	41.3	0.980
3-5cm	63	46.6		40.2	
>5cm	36	53.1		39.9	
Tumor location					
upper	9	35.6	0.348	11.1	0.055
middle	92	56.8		45.9	
lower	53	45.0		36.7	
Histologic grade					
G1	30	67.8	0.026	48.8	0.97
G2	101	51.9		41.3	
G3	23	26.9		26.4	
Invasive depth					
T1	12	91.7	0.021	80.2	0.008
T2	34	56.4		41.9	
T3	105	45.5		35.7	
T4	3	33.3		0.0	
Lymph node metastasis					
N0	84	64.3	0.000	50.5	0.001
N1	40	45.2		36.5	
N2	19	29.7		19.7	

N3	11	10.9		11.4	
pTNM-stage					
I	19	77.8	0.001	65.0	0.001
II	76	57.3		46.2	
III	59	34.5		25.1	

*, Log-rank test of Kaplan Meier method; P <0.05 was considered significant.

All patients underwent surgical treatment.

OS: overall survival

DFS: disease free survival

Supplementary Table 3. Univariate and multivariate analysis of factors associated with overall survival (OS) and disease-free survival (DFS) in the IHC cohort

Variables	Univariate analysis				Multivariate analysis			
	OS		DFS		OS		DFS	
	HR (95%CI)	*P*	HR (95%CI)	*P*	HR (95%CI)	*P*	HR (95%CI)	*P*
Age (>58 *vs* ≤58)	1.219(0.767 to 1.938)	0.402	1.191(0.785 to 1.807)	0.411				
Gender (Female *vs* Male)	1.515(0.845 to 2.717)	0.163	1.620(0.954 to 2.749)	0.074				
Tumor Size		0.886		0.981				
3-5cm vs ≤3cm	1.110(0.655 to 1.881)	0.697	0.995(0.620 to 1.597)	0.982				
>5cm vs ≤3cm	1.154(0.616 to 2.163)	0.654	1.048(0.599 to 1.833)	0.869				
Tumor Location		0.356		0.066				0.006
Middle vs Upper	0.859(0.339 to 2.177)	0.748	0.431(0.203 to 0.915)	0.028			0.276(0.125 to 0.611)	0.001
Lower vs Upper	1.230(0.473 to 3.196)	0.671	0.581(0.267 to 1.264)	0.171			0.296(0.127 to 0.692)	0.005
Histologic grade		0.032		0.108				
G2 vs G1	1.608(0.789 to 3.278)	0.191	1.295(0.723 to 2.320)	0.384				
G3 vs G1	2.908(1.267 to 6.674)	0.012	2.100(1.023 to 4.311)	0.043				
Lymph node metastasis		0.000		0.002		0.002		0.003
N1 vs N0	1.952(1.121 to 3.400)	0.018	1.792(1.090 to 2.945)	0.021	1.788(1.022 to 3.128)	0.042	1.928(1.150 to 3.233)	0.013
N2 vs N0	2.623(1.330 to 5.170)	0.005	2.561(1.407 to 4.664)	0.002	2.206(1.105 to 4.405)	0.025	2.537(1.346 to 4.782)	0.004
N3 vs N0	4.502(2.112 to 9.598)	0.000	2.975(1.432 to 6.183)	0.003	4.140(1.929 to 8.883)	0.000	3.175(1.483 to 6.797)	0.003
Invasive depth (T3+T4 vs T1+T2)	2.141(1.192 to 3.845)	0.011	1.771(1.075 to 2.918)	0.025				
pTNM-stage (III+IV vs I+II)	2.348(1.473 to 3.741)	0.000	2.130(1.402 to 3.238)	0.000				
CST1 (High vs Low) [a]	2.261(1.239 to 4.128)	0.008	1.864(1.121 to 3.098)	0.016	1.900(1.026 to 3.519)	0.041	1.671(0.976 to 2.861)	0.061

NOTE: Multivariate analysis, Cox proportional hazards regression model. Variables were adopted for their prognostic significance by univariate analysis.

[a] low, ≤7 positive fibroblast; high, >7 positive fibroblast.

Supplementary Table 4. The correlation between CST1 protein level and clinicopathological characteristics in ESCC

Variables	CST1[a]		chi-square value	*R*	*P**
	Low	High			
Age (year)					
≤58	23	58	0.109	0.027	0.742
>58	19	54			
Gender					
Male	31	84	0.023	0.012	0.880
Female	11	28			
Tumor size					
≤3cm	18	37	2.920	0.131	0.232
3-5cm	18	45			
>5cm	6	30			
Tumor location					
upper	4	5	3.689	0.154	0.158
middle	28	64			
lower	10	43			
Histologic grade					
G1	10	20	3.009	0.127	0.222
G2	29	72			
G3	3	20			
Invasive depth					
T1+T2	23	23	17.081	0. 333	0.000
T3+T4	19	89			
Lymph node metastasis					
N0	31	53	9.515	0.238	0.023
N1	8	32			
N2	2	17			
N3	1	10			
pTNM-stage					
I	13	6	21.818	0.350	0.000
II	21	55			
III	8	51			

* Chi-square Test; *P* value <0.05 was considered significant.

[a] low, ≤7 CST1-positive fibroblast cells; high, >7 CST1-positive fibroblast cells.
