# Supplementary material for: Integrated single-cell transcriptome analysis reveals heterogeneity of esophageal squamous cell carcinoma microenvironment
Source: Nat Commun. 2021 Dec 17;12:7335. doi: 10.1038/s41467-021-27599-5 (PMC8683407; doi:10.1038/s41467-021-27599-5)
Supplement: Supplementary file 2 — Description of Additional Supplementary Files [file 41467_2021_27599_MOESM2_ESM.docx]

**Description of Additional Supplementary Files**

File Name: Supplementary Data 1

Description: Differentially expressed genes of fibroblast, CD4^+^ T cell, CD8^+^ T cell, dendritic cell and monocyte, macrophage cell subsets. *P*-values and FDR-adjusted *P*-values account for multiple comparisons are calculated using MAST method based on hurdle model (see Method).

File Name: Supplementary Data 2

Description: Mean values for all significant interacting partners between myeloid cell-types with T cell subsets (the total mean of the individual partner average expression values in the corresponding interacting pairs of cell types).

File Name: Supplementary Data 3

Description: Genes associated with each diffusion component.

File Name: Supplementary Data 4

Description: Mean values for all significant interacting partners between specific fibroblast subsets with other cell-types (the total mean of the individual partner average expression values in the corresponding interacting pairs of cell types).
